# Misato Controls Mitotic Microtubule Generation by Stabilizing the Tubulin Chaperone Protein-1 Complex

**DOI:** 10.1016/j.cub.2015.05.033

**Published:** 2015-06-29

**Authors:** Valeria Palumbo, Claudia Pellacani, Kate J. Heesom, Kacper B. Rogala, Charlotte M. Deane, Violaine Mottier-Pavie, Maurizio Gatti, Silvia Bonaccorsi, James G. Wakefield

**Affiliations:** 1 Dipartimento di Biologia e Biotecnologie, Istituto Pasteur-Fondazione Cenci Bolognetti, Sapienza Università di Roma, Ple. A. Moro 5, 00185 Rome, Italy; 2 Biosciences, College of Life and Environmental Sciences, University of Exeter, Stocker Road, Exeter EX4 4QD, UK; 3 Proteomics Facility, Faculty of Medical and Veterinary Sciences, University of Bristol, Bristol BS8 1TD, UK; 4 Department of Statistics, University of Oxford, South Parks Road, Oxford OX1 3TG, UK; 5 Istituto di Biologia e Patologia Molecolari del CNR c/o Sapienza Università di Roma, 00185 Rome, Italy; 6 Institute of Molecular and Cellular Biology SD RAS, Novosibirsk 630090, Russia

## Abstract

Mitotic spindles are primarily composed of microtubules (MTs), generated by polymerization of α- and β-Tubulin hetero-dimers [1, 2]. Tubulins undergo a series of protein folding and post-translational modifications in order to fulfill their functions [3, 4]. Defects in Tubulin polymerization dramatically affect spindle formation and disrupt chromosome segregation. We recently described a role for the product of the conserved *misato* (*mst*) gene in regulating mitotic MT generation in flies [5], but the molecular function of Mst remains unknown. Here, we use affinity purification mass spectrometry (AP-MS) to identify interacting partners of Mst in the *Drosophila* embryo. We demonstrate that Mst associates stoichiometrically with the hetero-octameric Tubulin Chaperone Protein-1 (TCP-1) complex, with the hetero-hexameric Tubulin Prefoldin complex, and with proteins having conserved roles in generating MT-competent Tubulin. We show that RNAi-mediated in vivo depletion of any TCP-1 subunit phenocopies the effects of mutations in *mst* or the Prefoldin-encoding gene *merry-go-round* (*mgr*), leading to monopolar and disorganized mitotic spindles containing few MTs. Crucially, we demonstrate that Mst, but not Mgr, is required for TCP-1 complex stability and that both the efficiency of Tubulin polymerization and Tubulin stability are drastically compromised in *mst* mutants. Moreover, our structural bioinformatic analyses indicate that Mst resembles the three-dimensional structure of Tubulin monomers and might therefore occupy the TCP-1 complex central cavity. Collectively, our results suggest that Mst acts as a co-factor of the TCP-1 complex, playing an essential role in the Tubulin-folding processes required for proper assembly of spindle MTs.

## Highlights

• Misato interacts biochemically with the Tubulin Chaperone Protein-1 (TCP-1) complex
• Misato stabilizes the TCP-1 complex, possibly by filling its Tubulin-folding cavity
• Loss of Misato or TCP-1 complex subunits leads to similar mitotic phenotypes
• In the absence of Misato, Tubulin is unstable and unable to efficiently polymerize

## Teaser

Palumbo et al. show the conserved protein Misato interacts with the Tubulin Chaperone Protein-1 (TCP-1) complex in *Drosophila*. In the absence of Misato, TCP-1 subunit levels are reduced, functional Tubulin is unstable, and mitotic microtubule generation is compromised. The predicted structure of Misato suggests it acts as a molecular placeholder in the absence of Tubulin, stabilizing the TCP-1 complex.

## Results and Discussion

### Misato Biochemically Interacts with the Tubulin Chaperone Protein-1 Complex

We previously demonstrated that mutations in *misato* (*mst*) lead to frequent monopolar spindles (∼70%) with low microtubule (MT) density in *Drosophila* larval brains and that, upon MT regrowth after cold exposure, *mst* cells are primarily defective in kinetochore-driven MT generation [5].

**Figure 1:**
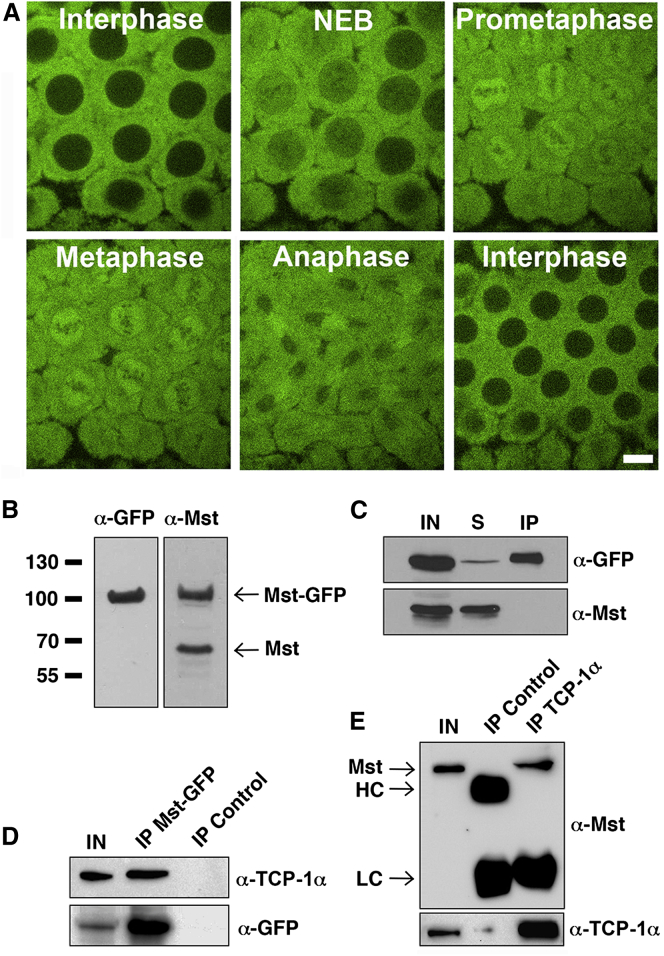
Mst Interacts with the TCP-1 Complex in the *Drosophila* Embryo (A) Selected frames from a time-lapse movie of a cycle 11 syncytial embryo expressing Mst-GFP. Note that Mst accumulates on the spindle. The scale bar represents 10 μm. (B) Western blots of extracts from Mst-GFP expressing embryos probed with anti-GFP and anti-Mst antibodies, showing that Mst-GFP and endogenous Mst are expressed at similar levels. (C) Immunoprecipitation of Mst-GFP from embryos using a GFP-TRAP-A-based affinity purification approach; GFP-TRAP-A binds Mst-GFP, but not endogenous Mst. IN, input (Mst-GFP embryo extract) (10%); S, supernatant (extract following incubation with GFP-TRAP-A beads); IP, immunoprecipitate (GFP-TRAP-A beads, post-incubation). (D) GFP-TRAP-A beads co-precipitate Mst-GFP and TCP-1α from embryo extracts (IP Mst-GFP). IN, input (Mst-GFP embryo extract) (10%); blocked agarose beads were used as a negative control (IP Control). (E) Western blot showing that anti-TCP-1α antibodies precipitate Mst from wild-type embryo extracts (IP TCP1-α); IgM was used as a negative control (IP Control). IN, input (wild-type embryo extract) (10%); HC and LC, IgG heavy and light chains, respectively.

**Figure 2:**
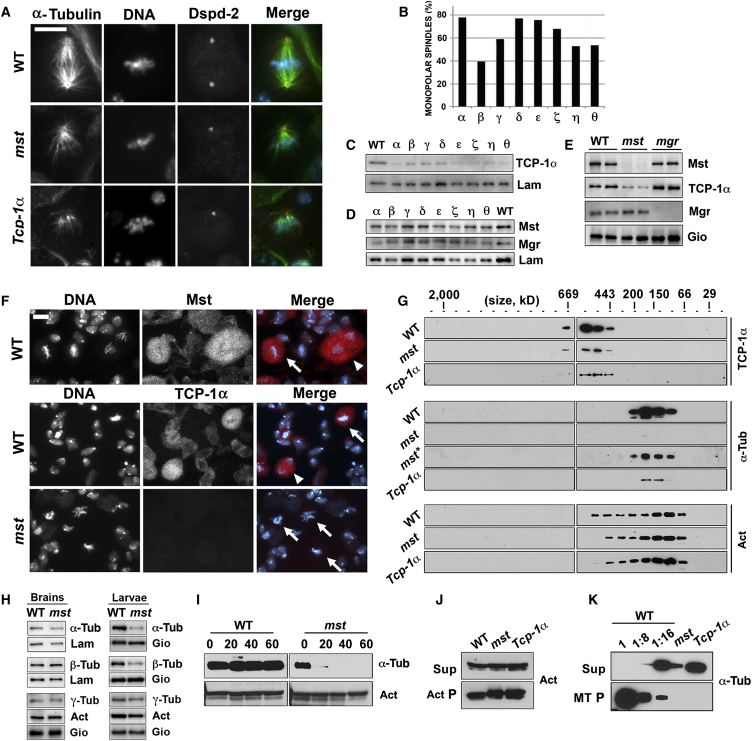
Mst Is Required for TCP-1 Complex Stability (A) *mst* and TCP-1α-depleted (*Tcp-1α*) neuroblast metaphases stained for Dspd-2 (red), α-Tubulin (green), and DNA (blue) exhibit monopolar spindles with low MT density. The scale bar represents 5 μm. (B) Frequencies of monopolar spindles observed in larval brains after RNAi-mediated depletion of TCP-1 subunits (indicated with Greek letters from α to θ). (C) Western blots of brain extracts depleted of TCP-1 subunits probed with anti-TCP-1α and anti-Lamin (Lam, loading control) antibodies. TCP-1α is reduced in all extracts. (D) Western blot of brain extracts depleted of TCP-1 subunits, probed for Mst, Mgr, and Lam. Mst and Mgr levels are similar to wild-type (WT). (E) Western blot from null *mst* and *mgr* mutant brains probed for Mst, TCP-1α, Mgr, and Giotto (Gio, loading control). TCP-1α levels are strongly reduced in *mst* extracts. (F) Localization of TCP-1α and Mst in larval brains. In WT brains, TCP-1α and Mst specifically accumulate in mitotic cells; in *mst* mutants, the TCP-1α signal is almost undetectable. Arrows and arrowheads point to metaphases and anaphases, respectively. The scale bar represents 5 μm. (G) Western blots of larval extracts, separated using size exclusion chromatography and probed for TCP-1α, α-Tubulin (α-Tub), and Actin (Act). Mst or TCP-1α loss reduces Tubulin, but not Actin, levels. Asterisk (^∗^) indicates overexposed WB to show Tubulin. (H) Western blots of larval brain and whole-larvae extracts showing total levels of α- and β-Tubulin, γ-Tubulin, and Actin in WT and *mst* mutants; α- and β-Tubulin are reduced in whole larvae, but not in brains. (I) Western blots of WT or *mst* larval extracts incubated at 27°C for 20, 40, or 60 min, probed for α-Tubulin and Actin. Tubulin is rapidly lost in *mst* extracts. (J) Western blot of an Actin sedimentation assay performed with WT, *mst*, and *Tcp-1α* RNAi larval extracts. Actin is able to polymerize in all extracts. (K) Western blot of a MT sedimentation assay. WT extracts were diluted 1:8 and 1:16 to control for reduced Tubulin levels in *mst* and *Tcp-1α* RNAi extracts. Tubulin is competent to polymerize in WT, but not in *mst* or *Tcp-1α* RNAi extracts.

To address the molecular function of Mst, we expressed a GFP-tagged variant in the *Drosophila* syncytial blastoderm embryo, which undergoes a series of rapid, synchronous nuclear divisions (Figure 1A; Movies S1 and S2). Expression of Mst-GFP fully rescued the lethality associated with the *mst*^*1*^ mutation and, under the control of the V32-GAL4 female germline-specific driver, Mst-GFP was expressed in embryos at similar levels to endogenous Mst (Figure 1B). Time-lapse experiments (Figure 1A; Movies S1 and S2) showed that Mst-GFP is excluded from interphase nuclei. Upon nuclear envelope breakdown, Mst accumulates in the region encompassed by the mitotic spindle but is excluded from centrosomes and astral MTs. By anaphase, Mst-GFP is most intense in the region corresponding to the central spindle MTs. This dynamic localization was confirmed by analysis of fixed embryos immunostained for α-Tubulin and Mst (Figure S1). In contrast, the analysis of brains expressing Mst-GFP under the control of an Actin-GAL4 driver (Figure S1 and Movie S3) and immunolocalization studies on fixed larval brains [5] (Figure 2F) failed to reveal Mst enrichment on the spindle. Thus, Mst is specifically associated with spindles of syncytial embryos.

To establish the biological process in which Mst functions, we sought to identify interacting partners. Syncytial embryos, which undergo 13 mitotic divisions predominantly in the absence of zygotic transcription, contain large amounts of mitotic proteins. We developed a pipeline based on GFP-TRAP-A affinity purification and mass spectrometry (AP-MS) combined with bioinformatics-based removal of non-specific contaminants, which allows the robust identification of GFP-bait protein interacting partners (see Supplemental Experimental Procedures). We undertook this procedure in triplicate for embryos expressing Mst-GFP. Incubation of 0–3 hr clarified embryo extracts with GFP-TRAP-A consistently depleted ∼95% of Mst-GFP, without affecting levels of untagged Mst, demonstrating that Mst is a monomer in the embryo (Figure 1C). Bioinformatics-based analysis of AP precipitates identified interactors, which suggested a core functional relationship with Mst. Table 1 shows a list of Mst-GFP interacting proteins from one of these experiments, after stringent filtering (for an extended list based on less stringent filtering, see http://www.thewakefieldlab.com/ms.html). A set of eight “top hit” proteins had similar MS scores and peptide coverages as Mst itself (∼1,300–3,000 and 70%–90%, respectively). These constitute all the subunits of the hetero-octameric Tubulin Chaperone Protein-1 (TCP-1) complex (Table 1). Also known as the Chaperonin Containing TCP-1 (CCT) or Tcp-1 Ring Complex (TRiC), the TCP-1 complex is an integral component of the Tubulin-folding pathway [3, 6, 7]. In this pathway, a complex termed Prefoldin [8, 9] initially interacts with newly synthesized α- and β-Tubulin, delivering them to the “donut”-shaped TCP-1 complex, which provides a suitable environment in which Tubulin can be correctly folded [3, 6–9]. A further set of conserved TCP-1 complex modulating proteins are additionally required, ensuring newly translated α- and β-Tubulin can be modified and incorporated into MTs [10]. The same pathway controls the proper folding of Actin and γ-Tubulin [11, 12].

All six subunits of the Prefoldin complex were also present in Mst-GFP AP precipitates (Table 1). Furthermore, two of the five remaining significant hits (Viaf and PDCD-5) are TCP-1 interactors. Viaf, a member of the Phosducin family, associates with the TCP-1 complex in flies [13], and its closest human homolog (PhLP-3) binds TCP-1, working antagonistically to Prefoldin [14]. Similarly, the human homolog of PDCD-5 binds to Phosducin and TCP-1β, interfering with the Tubulin-TCP-1 complex interaction [15].

The interaction between Mst and TCP-1α, the only subunit of the TCP-1 complex for which antibodies are available, was verified through reciprocal immunoprecipitation and western blotting in wild-type and Mst-GFP-expressing embryos (Figures 1D and 1E). Thus, the interaction between the TCP-1 complex and Mst reflects a normal in vivo interaction. Moreover, quantitative analysis of the MS data confirmed that similar quantities of all eight TCP-1 complex subunits and Mst were present in AP precipitates (Table 1). From this we infer that one molecule of Mst has the ability to interact with a single TCP-1 hetero-octameric complex. Notably, the Prefoldin subunits and the additional Tubulin-folding interactors were precipitated at ∼10- to 100-fold-lower amounts than the TCP-1 subunits. This most likely reflects transient ternary complexes that are formed between these proteins and the TCP-1 complex [8, 9, 13–15]. In summary, we demonstrate an in vivo biochemical relationship between Mst, the TCP-1 complex, and other Tubulin-folding pathway components.

### Depletion of the TCP-1 Complex Phenocopies the Loss of Mst and Merry-Go-Round

To investigate the functional relationship between Mst and its principal interactors, we compared the mitotic phenotypes elicited by depletion of either Mst, the *Drosophila* Prefoldin 3 subunit encoded by *merry-go-round* (*mgr*; [16, 17]), and the TCP-1 complex subunits. Available mutations in the genes encoding the TCP-1 complex subunits caused early embryonic lethality, preventing cytological analysis in larval brains. Similarly, in vivo RNAi, using UAS-RNAi lines and ubiquitous drivers, produced an early lethal phenotype. We thus generated flies carrying a suitable UAS-RNAi construct and the conditional tubGAL4-tubGAL80^ts^ driver, permitting time-restricted expression of this construct.

Brain preparations from larvae grown for 72 hr at 29°C, expressing UAS-RNAi constructs against the TCP-1 complex subunits, were immunostained for Tubulin and the centrosomal marker DSpd-2 [18] and compared with *mst* and *mgr* mutant brain preparations. Consistent with previous observations, *mst* and *mgr* mutant brains displayed a metaphase arrest phenotype and frequent polyploid cells [5, 16, 17, 19]. In addition, most *mst* and *mgr* prometaphase/metaphase figures showed monopolar spindles with reduced MT density (Figures 2A and S2). Importantly, RNAi against Tcp-1α caused an ∼80% reduction of the protein level and a mitotic phenotype indistinguishable from that elicited by *mst* mutations (Figures 2A–2C and S2). Moreover, RNAi against each of the other seven TCP-1 subunits also resulted in a very similar phenotype; we consistently observed high frequencies of monopolar spindles (ranging from 39.4% to 76.7%; n = 200 per each TCP-1 subunit) with strongly reduced MT density (Figures 2B, 2C, and S2).

To further investigate the functional relationship between Mst and the TCP-1 complex in spindle assembly, we analyzed MT regrowth after cold exposure in mitotic cells of TCP-1α-depleted brains. After cold-induced MT depolymerization, wild-type cells rapidly form new MTs, first from chromosomes and then from both chromosomes and centrosomes. The two MT populations merge within 5 min, giving rise to morphologically regular spindles ([5]; Figure S3). We previously showed that mutations in *mst* strongly reduce chromosome-driven MT regrowth, having little effect on MT regrowth from centrosomes [5]. Similarly, in TCP-1α-depleted brains exposed to cold and then returned at room temperature (RT), the majority of mitotic cells showed initial MT regrowth exclusively from the centrosomes; only after 10 min recovery did these cells show monopolar and bipolar spindles similar to those of untreated cells (Figure S3). Thus, the absence/reduction of Mst and TCP-1 subunits similarly affect spindle MT generation in larval neuroblasts, suggesting a functional link between Mst and the Tubulin-folding machinery.

### Mst Is Required for the Stability of the TCP-1 Complex and Efficient Tubulin Polymerization

We next sought to distinguish whether Mst is a substrate for the TCP-1 and Prefoldin complexes or whether it acts as a co-factor for the Tubulin-folding pathway. We first assessed the amount of Mst, Mgr, and TCP-1α in larval brain extracts depleted of each of these proteins. In extracts from RNAi larvae depleted of any TCP-1 subunit, the level of TCP-1α was always dramatically reduced (Figure 2C). These findings indicate that all TCP-1 complex components are required for TCP-1α stability and, consistent with previous results in mammalian cells [20], suggest that all TCP-1 subunits are mutually required for complex stability. However, their reduction did not affect the levels of either Mgr or Mst (Figure 2D). Remarkably, while levels of both Mst and TCP-1α were unaffected in brain extracts from *mgr* mutants, mutations in *mst*, although not affecting Mgr, caused a strong reduction in the levels of TCP-1α (Figure 2E). These findings were corroborated by immunolocalization experiments. Similar to Mst [5], TCP-1α specifically accumulated in the cytoplasm of mitotic cells of wild-type brains but was almost undetectable in dividing cells of *mst* mutant brains (Figure 2F).

To assess whether the low level of TCP-1α observed in *mst* mutants reflects a reduction of the entire TCP-1 complex, we subjected wild-type and *mst* mutant larval extracts to size exclusion chromatography, probing separated fractions for TCP-1α. The TCP-1α of *mst* mutants, although reduced in amount, eluted at ∼500–550 kD (corresponding to the predicted size of the *Drosophila* TCP-1 hetero-octameric complex) with a profile identical to wild-type TCP-1α (Figure 2G), indicating that Mst is required to stabilize the entire complex.

The TCP-1 complex in other experimental systems is essential for the correct folding of both Tubulin and Actin. However, while RNAi-mediated depletion of human TCP-1 complex subunits leads to a dramatic reduction in Tubulin levels, it has little effect on Actin [21]. To determine whether Mst depletion differentially affects Tubulin and Actin in *Drosophila*, we analyzed levels of α- and β-Tubulin, γ-Tubulin, and Actin in wild-type and *mst* mutant extracts from either brains or whole larvae. Although Tubulin levels were similar in wild-type and *mst* mutant brains, *mst* larvae displayed substantially reduced levels of α- and β-Tubulin compared to wild-type larvae (Figure 2H); this difference likely reflects variable susceptibilities of different tissues to loss of components of the Tubulin-folding pathway, as previously reported for *Drosophila* Mgr [17]. In contrast, both wild-type and *mst* mutant brains and larvae showed very similar levels of both γ-Tubulin and Actin. Moreover, size exclusion chromatography of wild-type, *mst* mutant extracts, or extracts of *Tcp1-α* RNAi larvae failed to reveal any effect on Actin level or size distribution profile, while confirming the reduction of α-Tubulin (Figure 2G).

To further investigate the specific loss and functionality of α- and β-Tubulin in *mst* mutant larvae, we performed Tubulin stability and MT sedimentation assays; we could not perform these experiments with isolated brains due to the difficulty in collecting enough tissue. First, by monitoring the amounts of Tubulin and Actin in extracts incubated at 27°C for varying times, we found that, while α-Tubulin levels remained unchanged in wild-type extracts over the course of 1 hr, α-Tubulin in *mst* mutant extracts was rapidly lost (Figure 2I). Next, we subjected extracts from wild-type, *mst*, and *Tcp1-α* RNAi larvae to in vitro MT and Actin sedimentation assays. We found that Actin polymerization was unaffected in *mst* or *Tcp1-α* RNAi extracts, although a consistent band-shift, possibly reflective of post-translational modification, was observed (Figure 2J). To control for the reduced concentration of α-Tubulin in *mst* and *Tcp1-α* RNAi larval extracts (Figure 2G), we performed the MT sedimentation assay with various diluted wild-type extracts. Even in wild-type extracts diluted 1:16, a proportion of α-Tubulin was able to pellet under polymerization conditions. In contrast, α-Tubulin in *mst* mutant larval extracts, similar to that present in *Tcp1-α* RNAi larvae, remained in the supernatant, suggesting an inability to polymerize (Figure 2K).

### The Role of Mst in Spindle MT Assembly

**Figure 3:**
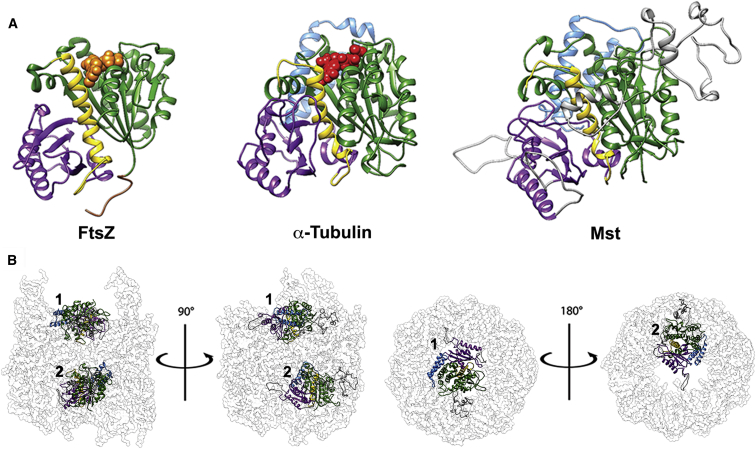
Structural Comparison of FtsZ, Tubulin, and Mst and Model of Mst in the TCP-1 Complex (A) Three-dimensional models of prokaryotic FtsZ (2VAW A), eukaryotic α-Tubulin (4I4T A), and Mst (Rosetta), annotated with Tubulin/FtsZ structural elements; bound nucleotides are drawn as spheres. Green indicates GTPase domain; yellow indicates helix 7; purple indicates activation domain; blue indicates C-terminal extension; orange indicates GDP; red indicates GTP; and gray indicates Mst loops. (B) Superimposition of Mst onto a partial model of Tubulin within the bovine TCP-1 complex (PDB: 2XSM). TCP-1 complex is represented as surface (white) and Mst as cartoons, colored as in (A). Top panels are front views of the complex, with two superimposed molecules of Mst (1 and 2) related by a 90° rotation around the depicted axis. Bottom panels are above and below views of the complex, respectively, showing only one superimposed Mst model (1 or 2), closer to the viewer, related by a 180° rotation around the depicted axis.

Our data demonstrate that the functions of Mst and the TCP-1 complex are integrally linked. Mst binds the complex and is required for its stability. Moreover, Mst loss causes a mutant phenotype indistinguishable from that elicited by loss of any TCP-1 subunit, and the biochemical properties of extracts depleted of either Mst or TCP1-α are identical. Together, these results indicate that Mst directly regulates TCP-1 complex structure and function. Mst was initially described as having primary structural motifs similar to those found in the Tubulin superfamily members [19, 22]. We therefore sought to determine whether Mst could be structurally related to Tubulins at the tertiary level. To do this, we composed a three-dimensional model of *Drosophila* Mst. Briefly, we identified sequences homologous to Mst in the α-, β-, and γ-Tubulin and FtsZ family of proteins, structurally aligned the available homologous structures, and merged Mst to this alignment. This final alignment was then used for comparative modeling using the Rosetta software suite (Figures 3 and S4; Supplemental Experimental Procedures).

The general secondary and tertiary structural elements found within both Tubulins and FtsZ map coherently onto Mst, strongly supporting the notion that Mst is a distant member of the Tubulin superfamily (Figure 3A). However, Mst contains additional stretches of amino acids that are predicted to be structurally disordered and that possibly affect both the nucleotide binding and oligomerization activities elicited by Tubulins (Figure 3A and S4). Upon superimposition of the Mst model onto the model of bovine Tubulin:TCP-1 complex [23], we found that Mst is capable of filling the internal cavity of the TCP-1 complex (Figure 3B). Although its additional loops exceed the cavity boundary, we hypothesize that the flexibility of these disordered protein stretches allows additional interactions with the TCP-1 complex. Therefore, although further work will be required to determine whether Mst does indeed sit within the TCP-1 complex cavity in vivo, our modeling is consistent with a scenario in which Mst stabilizes the TCP-1 complex in the absence of a substrate, through a Tubulin-like interaction.

Our study also highlights the existence of tissue-specific requirements for Tubulin folding and MT polymerization. We have shown that Mst associates with embryonic spindles, consistent with the report that *Drosophila* TCP-1 subunits co-sediment with MTs from early embryos [24]. However, Mst does not appear to be enriched in larval brain spindles ([5]; Figures 2F and S1). A possible explanation for this difference is that the rapid assembly of syncytial embryonic spindles [25] requires a high local concentration of assembly-competent Tubulin. A spindle-anchored folding machinery would, however, not be necessary in cells surrounded by a plasma membrane (such as brain cells), where sufficient folded Tubulin could be provided by increasing the intracellular concentration of the folding complexes in anticipation of mitosis.

Finally, the in vivo MT regrowth experiments presented here and previously [5] demonstrate that, in either *mst* mutant or *Tcp-1* RNAi background, centrosome-driven MT regrowth after cold treatment is less affected than chromosome-/kinetochore-induced regrowth. The simplest explanation is that, following cold-induced depolymerization, kinetochores and centrosomes nucleate MTs with different dynamics. Cold treatment removes the vast majority of cellular MTs, probably leaving only short, cold-resistant MT seeds at centrosomes. While rewarming of wild-type cells essentially “reboots” spindle assembly pathways, allowing both polymerization from centrosomes and ab-initio nucleation/polymerization from kinetochores, the reduced pool of assembly-competent Tubulin present in *mst* or *Tcp-1* mutant cells would be preferentially incorporated into the existing MT stubs at the centrosomes. An alternative hypothesis may reflect that the chromosome- and centrosome-driven MT formation pathways governing *Drosophila* cell division are at least in part under separate genetic control [5, 25, 26].

In summary, our study identifies Mst as a factor required for the stability of the TCP-1 complex, ultimately controlling the stability and polymerizing competency of α- and β-Tubulin within the fly. Mst is a conserved protein; future work will clarify whether it is a TCP-1 complex cofactor with a mitotic role in humans.

## Author Contributions

V.P. performed most of the experiments. C.P. and K.J.H. performed the biochemical and MS analyses, respectively. V.M.-P. generated Mst-GFP. Modeling of Mst was undertaken by K.B.R. under the supervision of C.M.D. J.G.W. performed the size exclusion chromatography. V.P., S.B., M.G., and J.G.W. conceived the experiments, analyzed the data, and wrote the paper.

## Figures and Tables

**Table 1:** List of Mst-GFP Interacting Proteins

Protein Name	Percentage Coverage	Number of Peptides	MW (kDa)	Score	Mean Area
TCP1-gamma	88.97	53	59	2,924	2.47E10
TCP1-zeta	82.74	46	58	2,188	2.14E10
TCP1-alpha	79.17	41	60	2,146	1.76E10
TCP1-beta	80.93	43	58	2,017	1.58E10
TCP1-eta	84.01	45	59	1,877	2.03E10
TCP1-delta	75.80	36	57	1,818	1.55E10
Misato	74.74	33	65	1,598	2.26E10
TCP1-theta	70.33	39	59	1,367	1.93E10
TCP1-epsilon	70.85	40	59	1,345	1.18E10
Prefoldin 5	74.41	32	55	579	3.06E9
Merry-go-round (Prefoldin 3)	35.05	9	22	115	1.91E9
CG7770 (Prefoldin 6)	38.40	5	14	77	6.60E8
l(3)01239 (Prefoldin 2)	58.04	9	16	75	5.14E8
CG10635 (Prefoldin 4)	20.29	2	16	54	1.14E8
CG13993 (Prefoldin 1)	29.37	6	15	52	5.26E8
Viaf	48.33	11	27	88	2.36E8
CG8378	26.00	9	67	67	1.51E8
CHIP	37.02	9	34	65	1.43E8
PDCD-5	57.89	6	15	53	2.05E8
CG5721	28.23	10	51	51	1.38E8

Proteins identified via mass spectrometry isolated from 0–3 hr Mst-GFP-expressing *Drosophila* embryo extracts after stringent filtering (see Supplemental Experimental Procedures). The proteins shown have MS scores of >50 and coverage percentages of >20%, respectively. Mean area corresponds to Top 3 Protein Quantification (T3PQ), the mean of the three highest abundance peptides identified for each protein. Mst is identified with a similar score and mean area to all eight subunits of the TCP-1 complex. All subunits of the Prefoldin complex are also co-precipitated, albeit at approximately 10-fold-lower abundance. Similar profiles were obtained for the TCP-1 subunits in the other two experiments.

## Supplemental Information

Document S1. Supplemental Experimental Procedures and Figures S1–S4

Movie S1. Dynamic Localization of Mst-GFP in *Drosophila* Syncytial Embryos Mst-GFP is excluded from nuclei prior to nuclear envelope breakdown (NEB). Upon NEB, the fusion protein re-localizes to the region of the spindle, but not to centrosomes or asters, persisting throughout metaphase. Mst-GFP is enriched in the region of the central spindle, which forms between the segregating chromosomes in anaphase/telophase.

Movie S2. Dynamic Co-localization of Mst-GFP and Rhodamine-Tubulin in *Drosophila* Syncytial Embryos During metaphase, Mst-GFP co-localizes with the mitotic spindle but is absent from centrosomes, which therefore appear red in the dual channel image. Mst-GFP co-localizes with the central spindle MTs that form between the segregating chromosomes in anaphase/telophase.

Movie S3. Dynamic Localization of Mst-GFP and His-RFP in Larval Brain Cells Within a cluster of larval brain cells, three are in mitosis, as assessed by chromosome condensation. The cell at the top of the field of view progresses from prometaphase to metaphase; the cell to the right is in metaphase throughout, while the cell in the bottom right undergoes anaphase. In all cells, Mst-GFP is exclusively cytoplasmic.

Document S2. Article plus Supplemental Information

## References

[1] Weisenberg R.C. (1972). Microtubule formation in vitro in solutions containing low calcium concentrations. Science.

[2] Walczak C.E., Heald R. (2008). Mechanisms of mitotic spindle assembly and function. Int. Rev. Cytol..

[3] Hartl F.U., Hayer-Hartl M. (2002). Molecular chaperones in the cytosol: from nascent chain to folded protein. Science.

[4] Westermann S., Weber K. (2003). Post-translational modifications regulate microtubule function. Nat. Rev. Mol. Cell Biol..

[5] Mottier-Pavie V., Cenci G., Vernì F., Gatti M., Bonaccorsi S. (2011). Phenotypic analysis of misato function reveals roles of noncentrosomal microtubules in Drosophila spindle formation. J. Cell Sci..

[6] Yaffe M.B., Farr G.W., Miklos D., Horwich A.L., Sternlicht M.L., Sternlicht H. (1992). TCP1 complex is a molecular chaperone in tubulin biogenesis. Nature.

[7] Yébenes H., Mesa P., Muñoz I.G., Montoya G., Valpuesta J.M. (2011). Chaperonins: two rings for folding. Trends Biochem. Sci..

[8] Vainberg I.E., Lewis S.A., Rommelaere H., Ampe C., Vandekerckhove J., Klein H.L., Cowan N.J. (1998). Prefoldin, a chaperone that delivers unfolded proteins to cytosolic chaperonin. Cell.

[9] Siegert R., Leroux M.R., Scheufler C., Hartl F.U., Moarefi I. (2000). Structure of the molecular chaperone prefoldin: unique interaction of multiple coiled coil tentacles with unfolded proteins. Cell.

[10] Willardson B.M., Howlett A.C. (2007). Function of phosducin-like proteins in G protein signaling and chaperone-assisted protein folding. Cell. Signal..

[11] Gao Y., Thomas J.O., Chow R.L., Lee G.H., Cowan N.J. (1992). A cytoplasmic chaperonin that catalyzes β-actin folding. Cell.

[12] Melki R., Vainberg I.E., Chow R.L., Cowan N.J. (1993). Chaperonin-mediated folding of vertebrate actin-related protein and gamma-tubulin. J. Cell Biol..

[13] Guruharsha K.G., Rual J.F., Zhai B., Mintseris J., Vaidya P., Vaidya N., Beekman C., Wong C., Rhee D.Y., Cenaj O. (2011). A protein complex network of Drosophila melanogaster. Cell.

[14] Stirling P.C., Srayko M., Takhar K.S., Pozniakovsky A., Hyman A.A., Leroux M.R. (2007). Functional interaction between phosducin-like protein 2 and cytosolic chaperonin is essential for cytoskeletal protein function and cell cycle progression. Mol. Biol. Cell.

[15] Tracy C.M., Gray A.J., Cuéllar J., Shaw T.S., Howlett A.C., Taylor R.M., Prince J.T., Ahn N.G., Valpuesta J.M., Willardson B.M. (2014). Programmed cell death protein 5 interacts with the cytosolic chaperonin containing tailless complex polypeptide 1 (CCT) to regulate β-tubulin folding. J. Biol. Chem..

[16] Gonzalez C., Sunkel C.E., Glover D.M. (1998). Interactions between mgr, asp, and polo: asp function modulated by polo and needed to maintain the poles of monopolar and bipolar spindles. Chromosoma.

[17] Delgehyr N., Wieland U., Rangone H., Pinson X., Mao G., Dzhindzhev N.S., McLean D., Riparbelli M.G., Llamazares S., Callaini G. (2012). Drosophila Mgr, a Prefoldin subunit cooperating with von Hippel Lindau to regulate tubulin stability. Proc. Natl. Acad. Sci. USA.

[18] Giansanti M.G., Bucciarelli E., Bonaccorsi S., Gatti M. (2008). Drosophila SPD-2 is an essential centriole component required for PCM recruitment and astral-microtubule nucleation. Curr. Biol..

[19] Miklos G.L., Yamamoto M., Burns R.G., Maleszka R. (1997). An essential cell division gene of Drosophila, absent from Saccharomyces, encodes an unusual protein with tubulin-like and myosin-like peptide motifs. Proc. Natl. Acad. Sci. USA.

[20] Kunisawa J., Shastri N. (2003). The group II chaperonin TRiC protects proteolytic intermediates from degradation in the MHC class I antigen processing pathway. Mol. Cell.

[21] Grantham J., Brackley K.I., Willison K.R. (2006). Substantial CCT activity is required for cell cycle progression and cytoskeletal organization in mammalian cells. Exp. Cell Res..

[22] Gurvitz A., Hartig A., Ruis H., Hamilton B., de Couet H.G. (2002). Preliminary characterisation of DML1, an essential Saccharomyces cerevisiae gene related to misato of Drosophila melanogaster. FEMS Yeast Res..

[23] Muñoz I.G., Yébenes H., Zhou M., Mesa P., Serna M., Park A.Y., Bragado-Nilsson E., Beloso A., de Cárcer G., Malumbres M. (2011). Crystal structure of the open conformation of the mammalian chaperonin CCT in complex with tubulin. Nat. Struct. Mol. Biol..

[24] Hughes J.R., Meireles A.M., Fisher K.H., Garcia A., Antrobus P.R., Wainman A., Zitzmann N., Deane C., Ohkura H., Wakefield J.G. (2008). A microtubule interactome: complexes with roles in cell cycle and mitosis. PLoS Biol..

[25] Hayward D., Metz J., Pellacani C., Wakefield J.G. (2014). Synergy between multiple microtubule-generating pathways confers robustness to centrosome-driven mitotic spindle formation. Dev. Cell.

[26] Bucciarelli E., Pellacani C., Naim V., Palena A., Gatti M., Somma M.P. (2009). Drosophila Dgt6 interacts with Ndc80, Msps/XMAP215, and gamma-tubulin to promote kinetochore-driven MT formation. Curr. Biol..

