# Misato Controls Mitotic Microtubule Generation by Stabilizing the TCP-1 Tubulin Chaperone Complex

**DOI:** 10.1016/j.cub.2015.07.035

**Published:** 2015-08-03

**Authors:** Valeria Palumbo, Claudia Pellacani, Kate J. Heesom, Kacper B. Rogala, Charlotte M. Deane, Violaine Mottier-Pavie, Maurizio Gatti, Silvia Bonaccorsi, James G. Wakefield

(Current Biology *25*, 1777–1783; June 29, 2015)

Our original article mistakenly used “Tubulin Chaperone Protein-1” as the basis for the abbreviation of the molecular chaperone complex TCP-1, including in the article title. We would like to clarify that the acronym TCP-1 is in fact derived from T-complex protein-1 (http://www.ncbi.nlm.nih.gov/gene/6950). As an alternative acronym for this same protein complex is the CCT (Chaperonin Containing TCP-1) complex, we have updated the nomenclature in the article to refer interchangeably to the protein complex that associated with Misato as “TCP-1/CCT.” The article title has also been updated (as shown here) to correct the erroneous “Tubulin Chaperone Protein-1” nomenclature. The authors sincerely apologize for the confusion, and for any inconvenience caused.

