# Supplementary material for: Misato Controls Mitotic Microtubule Generation by Stabilizing the Tubulin Chaperone Protein-1 Complex
Source: Curr Biol. 2015 Jun 29;25(13):1777–83. doi: 10.1016/j.cub.2015.05.033 (PMC4510148; doi:10.1016/j.cub.2015.05.033)
Supplement: Document S1. Supplemental Experimental Procedures and Figures S1–S4 [file mmc1.pdf]

Current Biology

Supplemental Information

Misato Controls Mitotic Microtubule Generation by Stabilizing the Tubulin Chaperone Protein-1 Complex

Valeria Palumbo, Claudia Pellacani, Kate J. Heesom, Kacper B. Rogala, Charlotte M. Deane, Violaine Mottier-Pavie, Maurizio Gatti, Silvia Bonaccorsi, and James G. Wakefield

Supplementary Figure S1

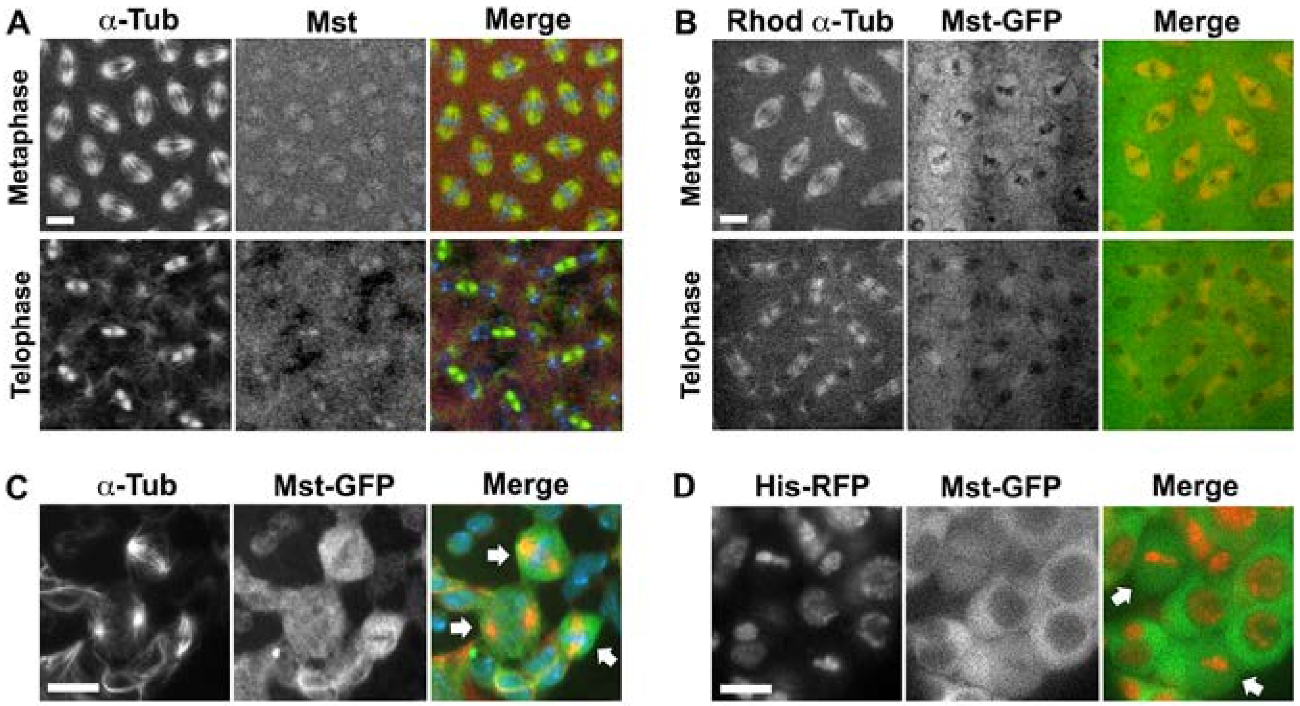

Supplementary Figure S1. Mst localisation in living and fixed embryos and brain cells. (A) Metaphase and telophase figures from cycle 12 fixed embryos stained for α-Tubulin (green), Mst (red) and DNA (blue). (B) Selected frames from a time-lapse movie of a cycle 11 embryo expressing Mst-GFP (green) and injected with Rhodamine-labelled α-Tubulin (Rhod α-Tub, red). In both live and fixed embryos, Mst co-localises with the region of the mitotic spindle, but not with centrosomes, during metaphase, and with the region of the central spindle during telophase. (C) Brain squashes from Mst-GFP expressing larvae immunostained for GFP and Tubulin. In merged figures, α-Tubulin is red, Mst-GFP is green and DNA is blue. (D) Single frame from a live preparation of larval neuroblasts expressing the red fluorescent protein-labeled histone H2AvD (His-RFP, red) and Mst-GFP (green). In both live and fixed brain cells, Mst-GFP is cytoplasmic during mitosis. Scale bar in A and B, 10 µm; in C and D, 5 µm. Related to Figure 1.

Supplementary Figure S2

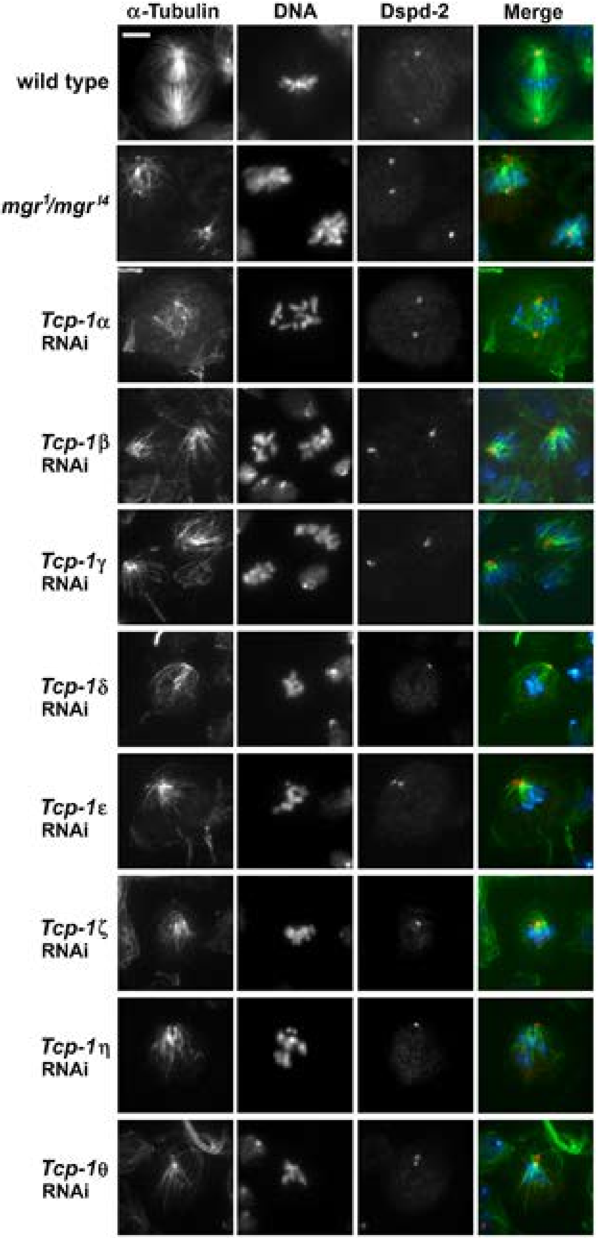

Supplementary Figure S2. ***mgr*** **mutant and TCP-1 complex subunit RNAi phenotypes**
Examples of mitotic spindles observed in brains from *mgr^1/mgr^{l4}* mutant larvae, and larvae expressing RNAi constructs against the indicated TCP-1 complex subunits. In merged figures, DNA is blue, α-Tubulin green, and DSpd-2 red. Scale bar, 5 μm. Related to Figure 2.

Supplementary Figure S3

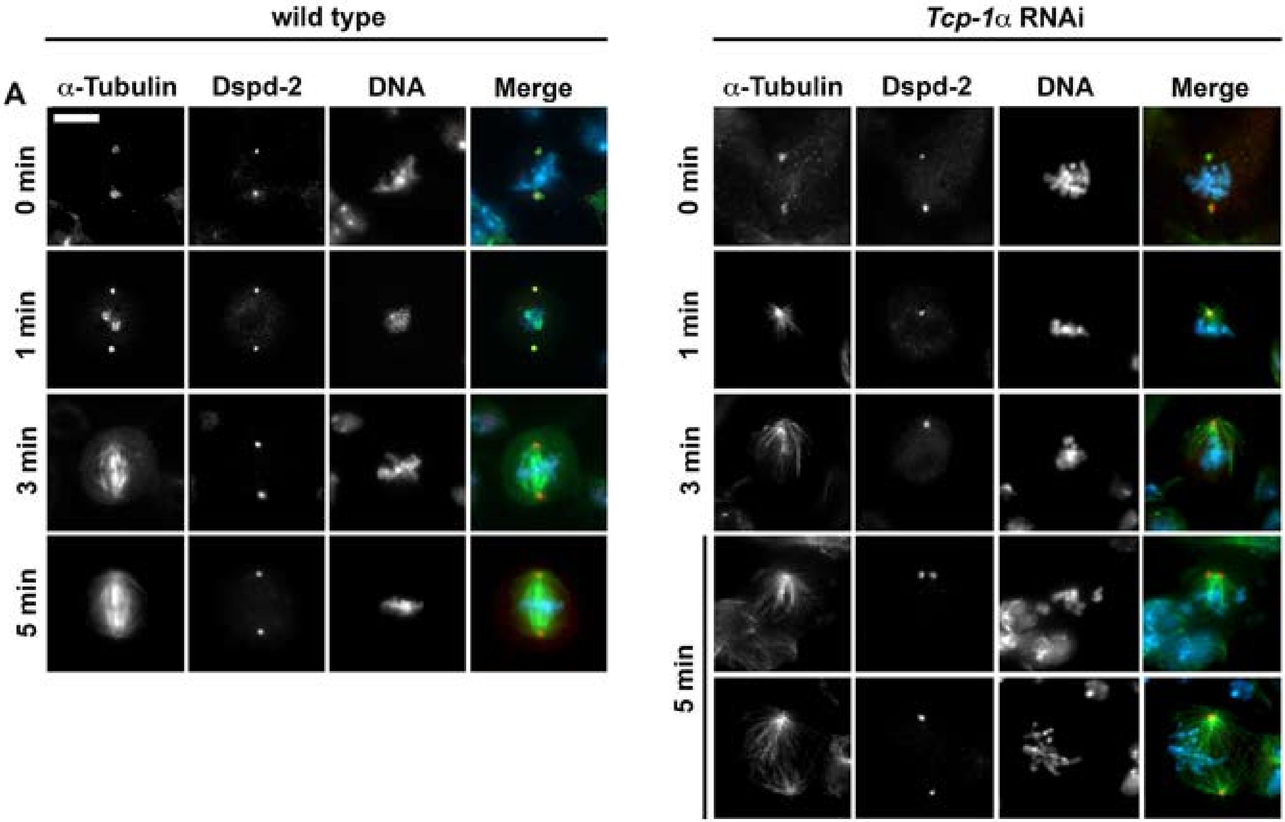

B

Genotype, time	# of cells	ch %	cn %	ch, cn %
wt, 1 min	90	62	2	36
wt, 3 min	70	40	0	60
wt, 5 min	75	0	0	100

C

Genotype, time	# of cells	ch %	cn %	ch, cn %
Tcp-1α RNAi, 1 min	60	8	80	12
Tcp-1α RNAi, 3 min	66	7	72	21
Tcp-1α RNAi, 5 min	50	6	66	28

Supplementary Figure S3. Microtubule regrowth after cold-induced depolymerisation
(A) After 50 min of cold treatment, MTs are completely depolymerized in both wild type and TCP1α-depleted brains. After 1 and 3 min recovery at room temperature, in wild type metaphase cells, microtubule (MT) regrowth occurs mainly near the chromosomes or from both the chromosomes and the centrosomes; after 5 min, most spindles are fully assembled. In TCP-1α-depleted cells, MT regrowth near the chromosomes is dramatically reduced at any time, and after 5 min most spindles only exhibit abnormally long centrosome-nucleated MTs. Cells were stained for DSpd-2 (red), Tubulin (green) and DNA (blue). Scale bar, 5 μm. (B, C) Frequencies of prometaphases/metaphases (P/M) from wild type (B) and TCP-1α-depleted brains (C) showing MT nucleation from the chromosomes only (ch), the centrosomes only (cn), or from both the chromosomes and the centrosomes (ch, cn). Related to Figure 2.

Supplementary Figure S4

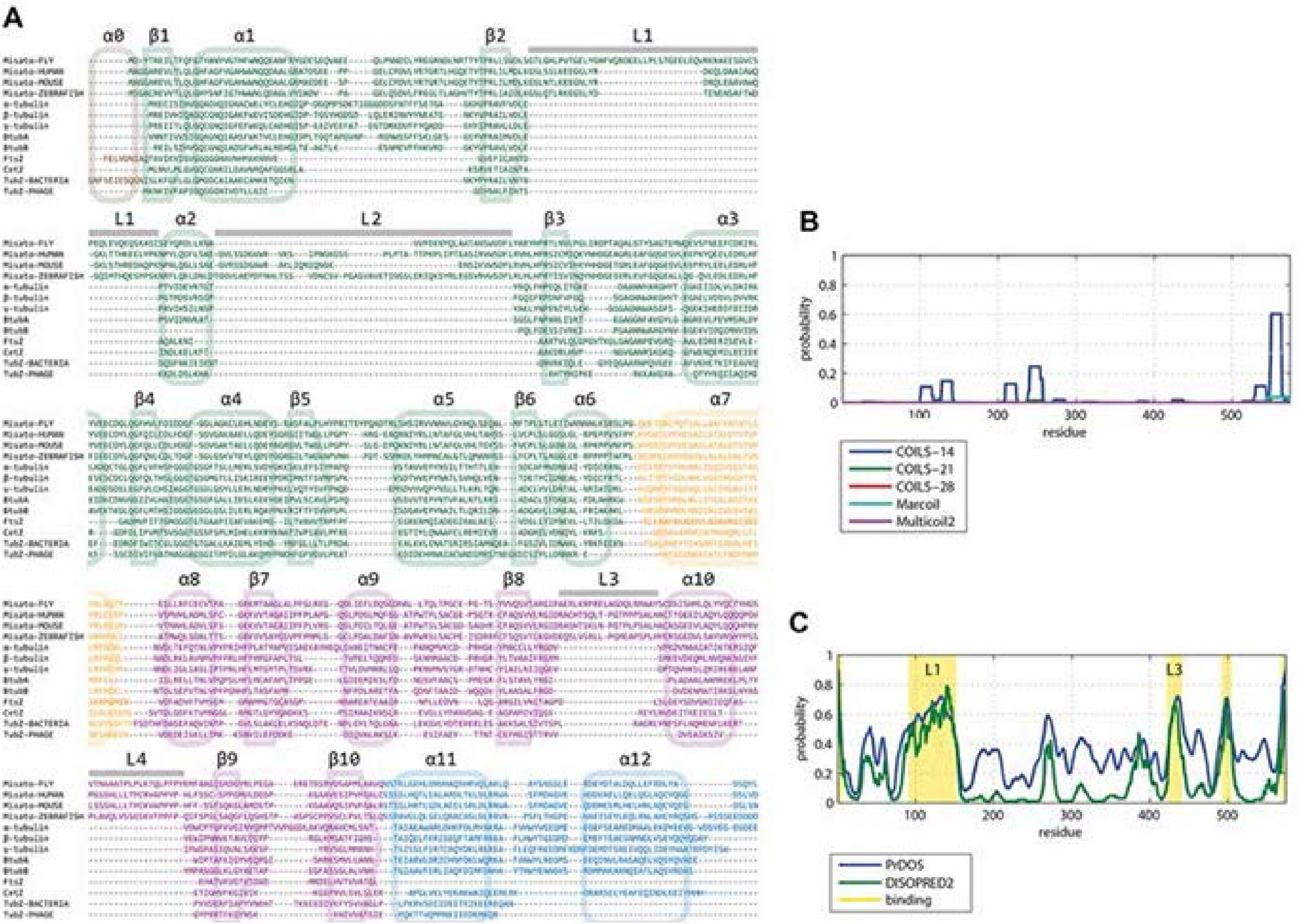

Supplementary Figure S4. Structural Bioinformatics Analysis of Misato. (A) Multiple sequence alignment of Mst and tubulin/FtsZ group of proteins. MSA7 was used for comparative modeling with RosettaCM (only representative sequences are shown; see Supplementary Experimental Procedures for the complete list), constructed by merging a structural alignment of Tubulin/FtsZ (MSA4) and the close homology alignment of Mst proteins (MSA6). Sequences used: Mst-FLY (RefSeq: NP_523435.1), Mst-HUMAN (RefSeq: NP_060586.2), Mst-MOUSE (GenBank: AAH08103.1), Mst-ZEBRAFISH (RefSeq: NP_956181.1), α-tubulin (PDB: 4I4T A), β-tubulin (PDB: 4I4T B), γ-tubulin (PDB: 3CB2 A), BtubA (PDB: 2BTO A), BtubB (PDB: 2BTQ B), FtsZ (PDB: 2VAW A), CetZ (PDB: 3ZID A), TubZ-BACTERIA (PDB: 4EI7 A), TubZ-PHAGE (PDB: 3V3T A). Colouring scheme: brown, N-terminal extensions; green, GTPase domain; yellow, helix 7; purple, activation domain; blue, C-terminal extension. Secondary structural elements are highlighted by corresponding boxes, with additional Mst loops marked by horizontal gray bars. (B) Coiled-coil formation propensity of Mst, predicted by COILS (window-14, blue; window-21, green; window-28, red; MTK matrix), Marcoil (cyan; MTK matrix), and Multicoil2 (magenta). (C) Disorder tendency predicted by PrDOS (blue) and DISOPRED2 (green). Yellow, transparent boxes signify potential binding sites in the disordered regions, some of which comprise additional loops of Mst: L1 and L3. Related to Figure 3.

SUPPLEMENTARY EXPERIMENTAL PROCEDURES

Drosophila strains

The inducible *mst*-GFP fly strain (carrying a pUASp-Mst-GFP element) was generated by cloning the full-length *mst* gene into the pPWG vector via pENTR/D-TOPO (Invitrogen). The plasmid was injected into *w1118* embryos by Bestgene Inc (Chino Hills, California), using standard procedures. Expression in the female germline was driven by V32-GAL4 (kindly provided by M. Bettencourt-Dias). For *in vivo* time-lapse imaging in larval brains, a strain was created containing both a P{H2AvD-RFP} insertion that constitutively expresses the red fluorescent protein-labelled histone H2AvD and the Mst-GFP transgene induced by the P{Act5C-GAL4}25FO1 driver insertion (FlyBase). The mst^{C27} and mst^{LB20} mutant alleles [S1] were obtained from Bloomington Stock Center. The *merry-go-round* alleles mgr^{1} and mgr^{J4} [S2, S3] were used to construct a late lethal *mgr* mutant genotype. TCP-1 complex subunits (TCP-1α-θ) RNAi fly strains (v34070, v108615, v106093, v106099, v109505, v109734, v 108585, v103905) were all obtained from the Vienna *Drosophila* RNAi center [S4]. The silencing was achieved by combining a single copy of *UAS-RNAi* transgene with a single copy of tub-GAL4, tub-GAL80^{ts} (kindly provided by T. Megraw; [S5]); cytological analysis was performed after 72 h of temperature shift from GAL4-suppressive (18°C) to permissive (29°C) temperature. Oregon-R strain was used as wild type. All flies were reared according to standard procedures and maintained at 25°C (unless otherwise specified). The genetic markers and special chromosomes are described in detail in FlyBase (http://www.flybase.org).

In vivo imaging

For *in vivo* time-lapse imaging of embryos, dechorionated 1-2 h old embryos expressing GFP-Mst were aligned in heptane glue on 22 × 50 mm coverslips, and covered with a 1:1 mixture of Halocarbon oil 700 and Halocarbon oil 27 (Sigma). For co-imaging of microtubules, Mst-GFP expressing embryos were injected with X-Rhodamine labelled tubulin (Cytoskeleton Inc.) at 5mg/ml in injection buffer (50mM K-HEPES pH 7.4, 50mM KCl) using an Eppendorf Inject Man NI 2 and Femtotips® II needles (Eppendorf). Imaging was performed using a Visitron Systems Olympus IX81 microscope equipped with a CSO-X1 spinning disk using a UPlanS APO 1.3 NA (Olympus) 60X objective. Five 1-µm slice stacks were acquired at a 400 ms exposure per slice, at a constant room temperature of 22°C. Image processing and analysis was performed using ImageJ to produce accumulated projections of the two 1-µm focal planes containing the highest signal intensity.

In vivo imaging of larval brains was carried out as described [S6]. Cells were examined using a calibrated Prior Proscan stepping motor, with an EM-CCD camera (Cascade II, Photometrics) connected to a spinning-disk confocal head (CarvII, Beckton Dickinson) mounted on an inverted microscope (Eclipse TE2000S, Nikon). The objective used was 100X (NA = 1.3). Image acquisition was controlled through the Metamorph software package (Universal Imaging, Downing Town, PA). Images were collected at 1-min intervals, and 7 fluorescence optical sections were captured at 1-µm z steps. Movies were created with Metamorph software; each fluorescent image shown is the maximum-intensity projection of all the sections.

Western blotting

Samples were run on standard SDS-PAGE gels, blotted and incubated with the following primary antibodies: mouse anti-Mst (1:5,000; Santa Cruz Biotech), mouse anti-α-Tubulin (1:10,000; Sigma, DM1A clone); rabbit anti-β-Tubulin (1:5,000; DSHB, E7 clone); rabbit anti-γ-Tubulin (1:2,000; Sigma, QG-17), mouse anti-GFP (1:10,000; Roche); mouse anti-Actin antibody (Sigma, AC-40 clone); rat anti-TCP-1α (1:1,000; Abcam); rabbit anti-Mgr (1:1,000; gift of D. Glover); mouse anti-Lamin Dm0 (1:5,000; DSHB, ADL101 clone); rabbit anti-Giotto (1:4,000; [S7]). For detection, the following HRP conjugated secondary antibodies were used: anti-mouse IgG (Sigma); anti-rabbit IgG (GE Healthcare), anti-rat IgG (GE Healthcare), all diluted 1:5-10,000. Samples were visualised by using enzyme-linked chemiluminescence and X-ray film, or imaged using ECL detection kit (GE Healthcare). Band intensities were quantified by densitometric analysis with Image Lab software (Bio-Rad).

GFP-TRAP-A isolation of Mst and immunoprecipitation

Flies expressing full length Mst-GFP under the control of an inducible promoter were crossed with the *V32-GAL4*/CyO strain. Batches of 0-3 h old embryos laid by cages of 1-10 day-old flies were dechorionated, weighted, flash frozen in N_2 (l) and stored at -80°C. For MS analysis, the following procedure was undertaken on three separate occasions: ~0.4 g of frozen embryos were homogenized in 1.5 ml of C buffer (50 mM HEPES [pH 7.4], 50 mM KCl, 1 mM $MgCl_2$, 1 mM EGTA, 0.1% IGEPAL CA-630, protease inhibitors (Roche)). Extract was clarified through centrifugation at 10,000 g for

10 min, 100,000 g for 30 min, and 100,000 g for a further 10 min. Clarified extract was incubated with 15 µl GFP-TRAP-A beads or blocked agarose beads (bab-20) (Chromotek) equilibrated in C Buffer for 2 h at 4°C. Mst-GFP/GFP-TRAP-A beads were then washed 4 times with ice-cold C buffer and stored at -20°C. For standard immunoprecipitations, between 0.1-0.2 g of frozen embryos were homogenized in proportional quantities of C buffer, clarified as above, and incubated with 30 μl of equilibrated Protein G sepharose beads (GE Healthcare) conjugated to anti-TCP-1α antibody for 2 h at 4°C , prior to extensive washes. Non-specific rat IgGs (Sigma) were used as co-IP negative controls.

Mass spectrometric analysis

Samples were run ~1 cm into the separating region of an SDS-PA gel, cut as a single slice and subjected to in-gel tryptic digestion using a DigestPro automated digestion unit (Intavis Ltd.). The resulting peptides were fractionated using a Dionex Ultimate 3000 nanoHPLC system in line with an LTQ-Orbitrap Velos mass spectrometer (Thermo Scientific). In brief, peptides in 1% (vol/vol) formic acid were injected onto an Acclaim PepMap C18 nano-trap column (Dionex). After washing with 0.5% (vol/vol) acetonitrile 0.1% (vol/vol) formic acid peptides were resolved on a 250 mm × 75 µm Acclaim PepMap C18 reverse phase analytical column (Dionex) over a 150 min organic gradient, using 7 gradient segments (1-6% solvent B over 1 min, 6-15% B over 58 min, 15-32% B over 58 min, 32-40% B over 3 min, 40-90% B over 1 min, held at 90% B for 6 min and then reduced to 1% B over 1 min) with a flow rate of 300 nl min^{-1}. Solvent A was 0.1% formic acid and Solvent B

was aqueous 80% acetonitrile in 0.1% formic acid. Peptides were ionized by nano-electrospray ionization at 2.1 kV using a stainless steel emitter with an internal diameter of 30 μm (Thermo Scientific) and a capillary temperature of 250°C. Tandem mass spectra were acquired using an LTQ- Orbitrap Velos mass spectrometer controlled by Xcalibur 2.1 software (Thermo Scientific) and operated in data-dependent acquisition mode. The Orbitrap was set to analyze the survey scans at 60,000 resolution (at m/z 400) in the mass range m/z 300 to 2000 and the top twenty multiply charged ions in each duty cycle selected for MS/MS in the LTQ linear ion trap. Charge state filtering, where unassigned precursor ions were not selected for fragmentation, and dynamic exclusion (repeat count, 1; repeat duration, 30 s; exclusion list size, 500) was used. Fragmentation conditions in the LTQ were as follows: normalized collision energy, 40%; activation q, 0.25; activation time, 10 ms; and minimum ion selection intensity, 500 counts.

The raw data files were processed and quantified using Proteome Discoverer software v1.2 (Thermo Scientific) and searched against the dmel-all-translation-r5.47 database using the SEQUEST (Ver. 28 Rev. 13) algorithm. Peptide precursor mass tolerance was set at 10ppm, and MS/MS tolerance was set at 0.8Da. Search criteria included carbamidomethylation of cysteine (+57.0214) as a fixed modification and oxidation of methionine (+15.9949) as a variable modification. Searches were performed with full tryptic digestion and a maximum of 1 missed cleavage was allowed. The reverse database search option was enabled and all peptide data was filtered to satisfy false discovery rate (FDR) of 5%.

Bioinformatics filtering of MS data

For stringent filtering, MS results were filtered by removing protein IDs with (i) single peptide hits, (ii) <20% peptide:protein coverage and (iii) overall MS Scores of <50. Through our on-going studies, we have produced a database of MS data accumulated from eight independent control GFP-TRAP-A experiments, each using extracts from ~0.4 g 0-3 h embryos expressing GFP-fusions to proteins in which a bait protein was not precipitated (i.e. negative controls). A spread-sheet incorporating these data was used to produce a list of false-positive protein IDs and their associated highest overall MS Score. Filtered Mst-GFP AP-MS results were cross-referenced against this database. Any protein ID that was either not identified in negative control list or was identified in negative controls with MS Scores of at least 4 fold less than in Mst-GFP was kept, while all other protein IDs were discarded. Each of the three replicate experiments produced similar results, with all TCP-1 complex subunits identified with high confidence in each case. The result of this stringent combined filtering/false-positive for one of these datasets is shown in Table 1. A less stringent filtering of this dataset (discarding only <10% peptide:protein coverage and (iii) overall MS Scores of <30) can be found on the Wakefield lab web-site (www.thewakefieldlab.com/ms.html), demonstrating the validity of the stringent analysis in maximising the confidence of the interaction dataset.

Cytology

For immunofluorescence experiments, 1-2 h old embryos were collected at 25ºC on agar plates and dechorionated in 50% bleach. After removal of the

vitelline membrane in a mixture of methanol and heptane (1:1), embryos were fixed for 30 min in 3.7% formaldehyde in PBS under gentle agitation at room temperature and blocked for 1 hour in 0.3% Triton X-100 PBS with 3% BSA before staining. For double immuno-staining of Mst and spindle MTs, embryos were first incubated O/N at 4°C with a monoclonal anti-Mst antibody (1:50; Santa Cruz Biotech), then for 1 hour at room temperature with Rhodamine-conjugated anti-mouse IgG (1:50; Jackson Laboratories) and finally with a FITC-conjugated anti α-Tubulin antibody (1:150, Sigma) for 2 hours at room temperature. Preparations were stained with TOTO-3 DNA dye (1:1,000; Life Technologies) for 10 min at room temperature and then mounted in Vectashield medium H-1000 (Vector Laboratories). Confocal analysis was performed with a laser scanning inverted microscope Zeiss LSM 780 (Zeiss, Oberckochen, Germany) equipped with a 63X/1.4 Oil Plan-Apochromat objective. Image acquisition and processing were achieved using the Zeiss Efficient Navigation (ZEN) software. The images shown are the maximum-intensity projections of optical sections acquired at 0.5 μm.

Fixation and immuno-staining of larval brains was performed as previously described [S8]. Brain squashes were incubated overnight at 4°C with the following primary antibodies: rabbit anti DSpd-2 [S9], monoclonal anti-α-Tubulin (1:1,000; Sigma-Aldrich), rat anti-TCP-1α (Abcam) and rabbit anti-GFP (1:300; Torrey Pines Biolabs), which were detected by 1 hour incubation at room temperature with fluorescein isothiocyanate (FITC)-conjugated anti-mouse IgG+IgM (1:20; Jackson Laboratories), CY3-coniugated anti-rabbit IgG (1:300; Invitrogen), FITC-coniugated anti-rabbit IgG (1:50; Jackson Laboratories) and FITC-conjugated anti-rat IgG (1:20; Roche). Immunostained

preparations were mounted in Vectashield medium H-1200 (Vector Laboratories) containing the DNA dye DAPI, and examined with a Zeiss Axioplan fluorescence microscope equipped with a CCD camera (Photometrics CoolSnap HQ).

Microtubule regrowth assay

Larval brains were dissected in 0.7% NaCl and placed on ice for 50 min. After cold-induced depolymerisation, brains were either immediately fixed as previously described [S8], or placed at 22°C for 1, 3, 5 min and then fixed. All preparations were immunostained for α-Tubulin and Dspd-2 as described above.

Size exclusion chromatography

Analytical gel filtration chromatography was carried out using a Superose 6 10/300 GL column (24 ml bed volume; GE Healthcare) attached to an AKTA pure 25L system (Life Tech). Columns were run with C Buffer (without protease inhibitors) at 4°C at a flow of 0.5 mL/min. The following globular molecular weight standards were used to calibrate the column, at 10mg/ml: Dextran 2000 (2 MDa), thyroglobulin (669 kDa), apoferritin (440 kDa), alcohol dehydrogenase (DH) (150 kDa), albumin (67 kDa), carbonic anhydrase (29 kDa) (Sigma). The logarithm of the molecular weight (LogMW) of the standard proteins obtained from three independent runs was plotted against the elution volume (mL). 60 3rd instar larvae (Oregon-R, *mst* mutant and *Tcp1-α* RNAi) were flash frozen in N_2 (l) and stored at -80°C. Samples were ground using a pestle and mortar, suspended in 1 ml cold C buffer and spun at 65,000 rpm in

a TLA100 rotor in a Beckman Ultracentrifuge at 4°C for 30 min. Clarified supernatants were assessed for protein concentration using a nanodrop. Concentrations were: Oregon-R, 15.5 mg/ml; *mst*, 13.8 mg/ml; *Tcp-1*α RNAi, 13.6 mg/ml). 500 µl of clarified supernatant of each genotype was run sequentially through the column and 0.5 ml fractions collected. Aliquots were prepared for analysis by SDS-PAGE and western blotting by the addition of 6X protein sample buffer (PSB).

Larval MT sedimentation and tubulin stability assays

For the tubulin stability assay, batches of 30 control (Oregon-R) or *mst* 3rd instar larvae, flash frozen in N_2 (l) and stored at -80°C, were ground using a pestle and mortar, suspended in 500 µl of cold C buffer and spun at 65,000 rpm in a TLA100 rotor in a Beckman Ultracentrifuge at 4°C for 30 min. Immediately following centrifugation, 50 µl of extract was added to 10 µl of 6X PSB, while the remainder of the extract was placed in a water bath at 25°C. After 20 min, 40 min and 60 min, further 50 µl samples were added to 10 µl 6X PSB. Samples were run on standard SDS-PAGE gels and analysed by Western Blotting.

For MT sedimentation assays, clarified supernatants were generated from 30 frozen control or *mst* 3rd instar larvae as above. 150 µl of each clarified supernatant was incubated at 25°C for 20 min in the presence of 1 mM GTP, to stimulate MT polymerisation. Samples were carefully loaded over a two-volume cushion of C buffer + 40% glycerol and centrifuged at 65,000 rpm at 25°C for 12 min. 50 µl of supernatants were added to 10 µl of 6X PSB. The remaining supernatant was carefully removed, the interface between

supernatant and glycerol cushion washed with 100 µl of C Buffer, and all sample removed, being careful not to disturb any pellet. 50 µl of 1X PSB was added to the bottom of the tube, and pipetted to resuspend any pellet. This assay was undertaken on three separate occasions and produced qualitatively similar results in all cases, though the amount of Tubulin present in the *mst* mutant extracts, in relation to controls, varied, presumably due to variation in Tubulin degradation.

Homology search and MSA building

The homology search was performed on the Protein Data Bank database using HHsearch [S10], with HHblits [S11] MSA generation method (global alignment, secondary structure scoring, MSA1). Strong hits for models in the Tubulin and FtsZ families, derived from X-ray crystallography, with sequence coverage of over 60%, and resolution better than 3.2Å, were divided into individual domains (GTPase domain - MSA2 and activation domain - MSA3), and structurally realigned with POSA [S12] to account for flexibility of different conformational states. Using helix 7 as the overlapping region between the domains, the alignments (MSA2 and 3) were merged and manually refined (MSA4). Furthermore, we used HMMER (jackhammer) [S13] to find close homologues of *D. melanogaster* Mst in both invertebrates and vertebrates (NCBI nr database, MSA5). Hits covering more than 80% of the sequence were realigned using MAFFT [S14] (G-INS-i method, MSA6). In order to accurately relate Mst to tubulin/FtsZ, we merged the structural alignment of tubulin/FtsZ (MSA4) and the close homology alignment of Mst proteins (MSA6) using MAFFT [S15] (E-INS-i method, MSA7) while preserving both

alignments in their respective profiles.

Comparative modelling

Modelling of Mst was performed with Rosetta 3.5 [S16] comparative modelling protocol [S17]. The calculation was guided by the multiple sequence alignment (MSA7, Figure S4) to homologous proteins of known structure, minimising the conformational search space by providing a scaffold for protein backbone modelling. The structural templates used were: Tubulins: 4I4T A, 4I4T B, 3CB2 A; Bacterial tubulins (Btub): 2BTO A, 2BTQ B; CetZ: 3ZID A, 4B45 A, 4B46 A**;** FtsZ: 2R75 1, 2VXY A, 4M8I A, 1RQ2 A, 1OFU A, 2VAW A, 2VAP A, 1W5F A/B (GTPase domain from A, and activation domain from B); Bacterial TubZ: 3M89 A, 4EI7 A**;** Bacteriophage TubZ: 3V3T A, 3ZBQ A, 3R4V A. RosettaCM uses hybridisation protocol which allows for simultaneous sampling of multiple conformations featured in various protein templates. Regions considerably divergent from templates or those involving insertions or 'loops', were modeled *de novo* with Rosetta loop-building protocol. In order to exhaustively sample the local conformational space of the loops exhaustively, we calculated 2000 decoys: 400 decoys for each of the 5 best scoring templates (PDB ID: 2BTO_A, 2BTQ_B, 3CB2_A, 4I4T_A, 4I4T_B), which were further refined using Rosetta all-atom scoring function, energetically minimising the entire protein. The decoys were subsequently clustered with CALIBUR [S18] to identify structurally similar models.

Other bioinformatics and structural calculations

Coiled-coil predictions were carried out using COILS [S19] (windows: 14, 21

and 28; MTK matrix), Marcoil [S20] (MTK matrix) and Multicoil2 [S21] (Figure S5). Protein disorder was estimated using PrDOS [S22] and DISOPRED2 [S23], which was also used to predict propensity of disordered regions to bind globular proteins. Protein structure figures were prepared in Chimera [S24], which was also used to calculate superimpositions (MatchMaker tool).

SUPPLEMENTARY REFERENCES

S1. Miklos, G.L., Yamamoto, M., Burns, R.G., and Maleszka, R. (1997). An essential cell division gene of *Drosophila*, absent from *Saccharomyces*, encodes an unusual protein with tubulin-like and myosin-like peptide motifs. Proc. Natl. Acad. Sci. U S A. 94, 5189-94.

S2. Ripoll, P., Casal, J., and Gonzalez, C. (1987). Towards the genetic dissection of mitosis in *Drosophila*. BioEssays. 7: 204-210.

S3. Delgehyr, N., Wieland, U., Rangone, H., Pinson, X., Mao, G., Dzhindzhev, N.S., McLean, D., Riparbelli, M.G., Llamazares, S., Callaini, G., et al. (2012). *Drosophila* Mgr, a Prefoldin subunit cooperating with von Hippel Lindau to regulate tubulin stability. Proc. Natl. Acad. Sci. U S A. 109, 5729-34.

S4. Dietzl, G., Chen, D., Schnorrer, F., Su, K.C., Barinova, Y., Fellner, M., Gasser, B., Kinsey, K., Oppel, S., Scheiblauer, S., et al. (2007). A genome-wide transgenic RNAi library for conditional gene inactivation in *Drosophila*. Nature. 448, 151-6.

S5. Suster, M.L., Seugnet, L., Bate, M., and Sokolowski, M.B. (2004). Refining GAL4-driven transgene expression in *Drosophila* with a GAL80 enhancer-trap. Genesis. 39, 240-5.

S6. Rahmani, Z., Gagou, M.E., Lefebvre, C., Emre, D., and Karess, R.E. (2009). Separating the spindle, checkpoint, and timer functions of BubR1. J. Cell Biol. 187, 597-605.

S7. Giansanti, M.G., Bonaccorsi, S., Kurek, R., Farkas, R.M., Dimitri, P., Fuller, M.T., and Gatti, M. (2006). The class I PITP giotto is required for

Drosophila cytokinesis. Curr. Biol. 16, 195-201.

S8. Bonaccorsi, S., Giansanti, M.G., and Gatti, M. (2000). Spindle assembly in *Drosophila* neuroblasts and ganglion mother cells. Nat. Cell Biol. 2, 54-6.

S9. Giansanti, M.G., Bucciarelli, E., Bonaccorsi, S., and Gatti, M. (2008). *Drosophila* SPD-2 is an essential centriole component required for PCM recruitment and astral-microtubule nucleation. Curr. Biol. 18, 303-9.

S10. Söding, J. (2005). Protein homology detection by HMM-HMM comparison. Bioinformatics. 21, 951-60.

S11. Remmert, M., Biegert, A., Hauser, A., and Söding, J. (2011). HHblits: lightning-fast iterative protein sequence searching by HMM-HMM alignment. Nat. Methods. 9, 173-5.

S12. Li, Z., Natarajan, P., Ye, Y., Hrabe, T., and Godzik, A. (2014). POSA: a user-driven, interactive multiple protein structure alignment server. Nucleic Acids Res. 42, W240-5.

S13. Finn, R.D., Clements, J., and Eddy, S.R. (2011). HMMER web server: interactive sequence similarity searching. Nucleic Acids Res. 39, W29-37.

S14. Katoh, K., and Standley, D.M. (2013). MAFFT multiple sequence alignment software version 7: improvements in performance and usability. Mol Biol Evol. 30, 772-80.

S15. Katoh, K., and Frith, M.C. (2012). Adding unaligned sequences into an existing alignment using MAFFT and LAST. Bioinformatics. 28, 3144-6.

S16. Leaver-Fay, A., Tyka, M., Lewis, S.M., Lange, O.F., Thompson, J., Jacak, R., Kaufman, K., Renfrew, P.D., Smith, C.A., Sheffler, W., et al. (2011). ROSETTA3: an object-oriented software suite for the simulation and design of macromolecules. Methods Enzymol. 487, 545-74.

S17. Thompson, J., and Baker, D. (2011). Incorporation of evolutionary information into Rosetta comparative modeling. Proteins. 79, 2380-8.

S18. Li, S.C., and Ng, Y.K. (2010). Calibur: a tool for clustering large numbers of protein decoys. BMC Bioinformatics, 11:25.

S19. Lupas, A., Van Dyke, M., and Stock, J. (1991). Predicting coiled coils from protein sequences. Science. 252, 1162-4.

S20. Delorenzi, M., and Speed, T. (2002). An HMM model for coiled-coil domains and a comparison with PSSM-based predictions. Bioinformatics. 18, 617-25.

S21. Trigg, J., Gutwin, K., Keating, A.E., and Berger, B. (2011). Multicoil2: predicting coiled coils and their oligomerization states from sequence in the twilight zone. PLoS One. 6, e23519.

S22. Ishida, T., and Kinoshita, K. (2007). PrDOS: prediction of disordered protein regions from amino acid sequence. Nucleic Acids Res. 35, W460-4.

S23. Ward, J.J., McGuffin, L.J., Bryson, K., Buxton, B.F., and Jones, D.T. (2004). The DISOPRED server for the prediction of protein disorder. Bioinformatics. 20, 2138-9.

S24. Pettersen, E.F., Goddard, T.D., Huang, C.C., Couch, G.S., Greenblatt, D.M., Meng, E.C., and Ferrin, T.E. (2004). UCSF Chimera--a visualization system for exploratory research and analysis. J Comput Chem. 25, 1605-12.
