# Supplementary figures and images for: Misato Controls Mitotic Microtubule Generation by Stabilizing the Tubulin Chaperone Protein-1 Complex
Source: Curr Biol. 2015 Jun 29;25(13):1777–83. doi: 10.1016/j.cub.2015.05.033 (PMC4510148; doi:10.1016/j.cub.2015.05.033)

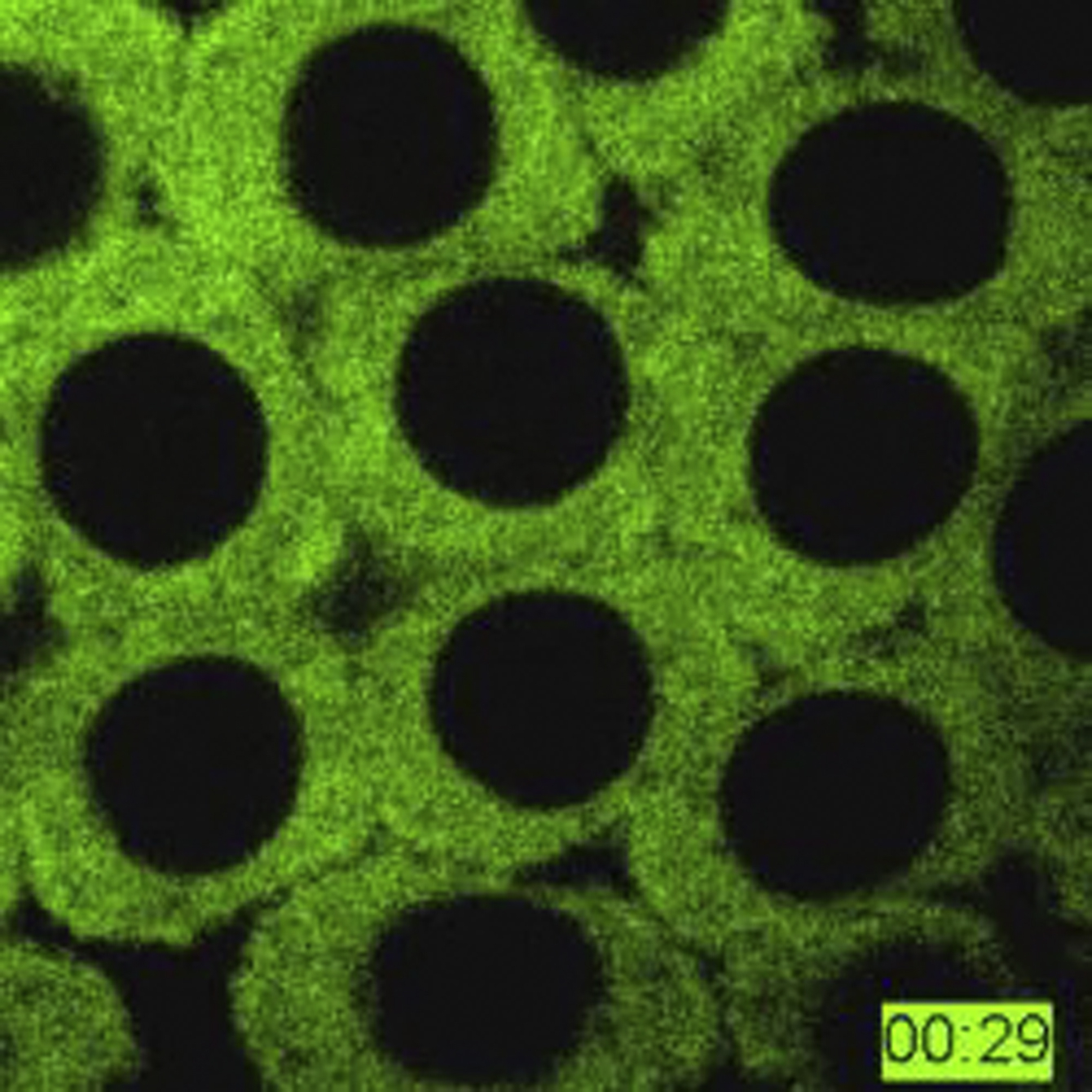

Supplement: Movie S1. Dynamic Localization of Mst-GFP in Drosophila Syncytial Embryos — Mst-GFP is excluded from nuclei prior to nuclear envelope breakdown (NEB). Upon NEB, the fusion protein re-localizes to the region of the spindle, but not to centrosomes or asters, persisting throughout metaphase. Mst-GFP is enriched in the region of the central spindle, which forms between the segregating chromosomes in anaphase/telophase. [file mmc2.jpg]

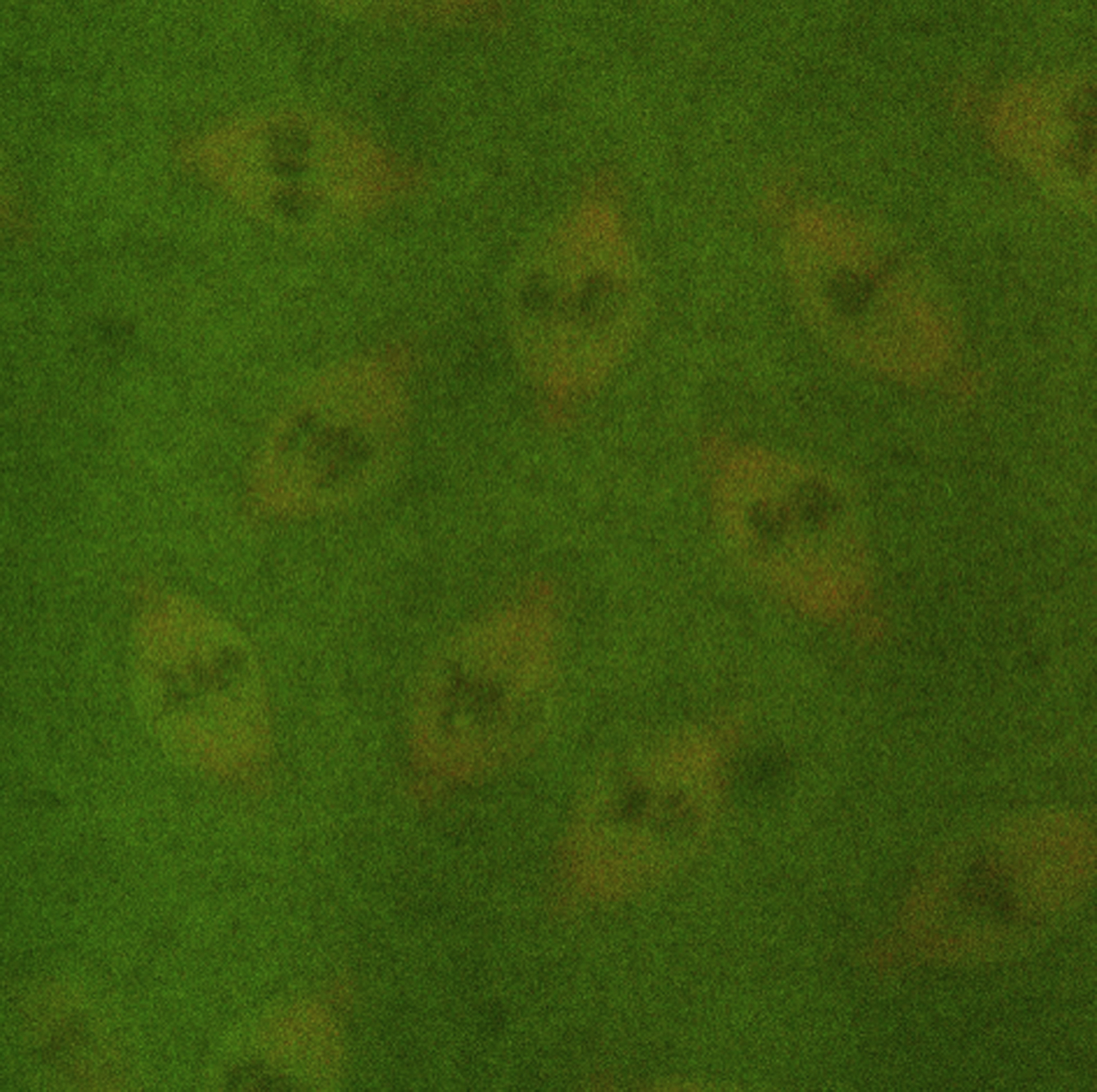

Supplement: Movie S2. Dynamic Co-localization of Mst-GFP and Rhodamine-Tubulin in Drosophila Syncytial Embryos — During metaphase, Mst-GFP co-localizes with the mitotic spindle but is absent from centrosomes, which therefore appear red in the dual channel image. Mst-GFP co-localizes with the central spindle MTs that form between the segregating chromosomes in anaphase/telophase. [file mmc3.jpg]

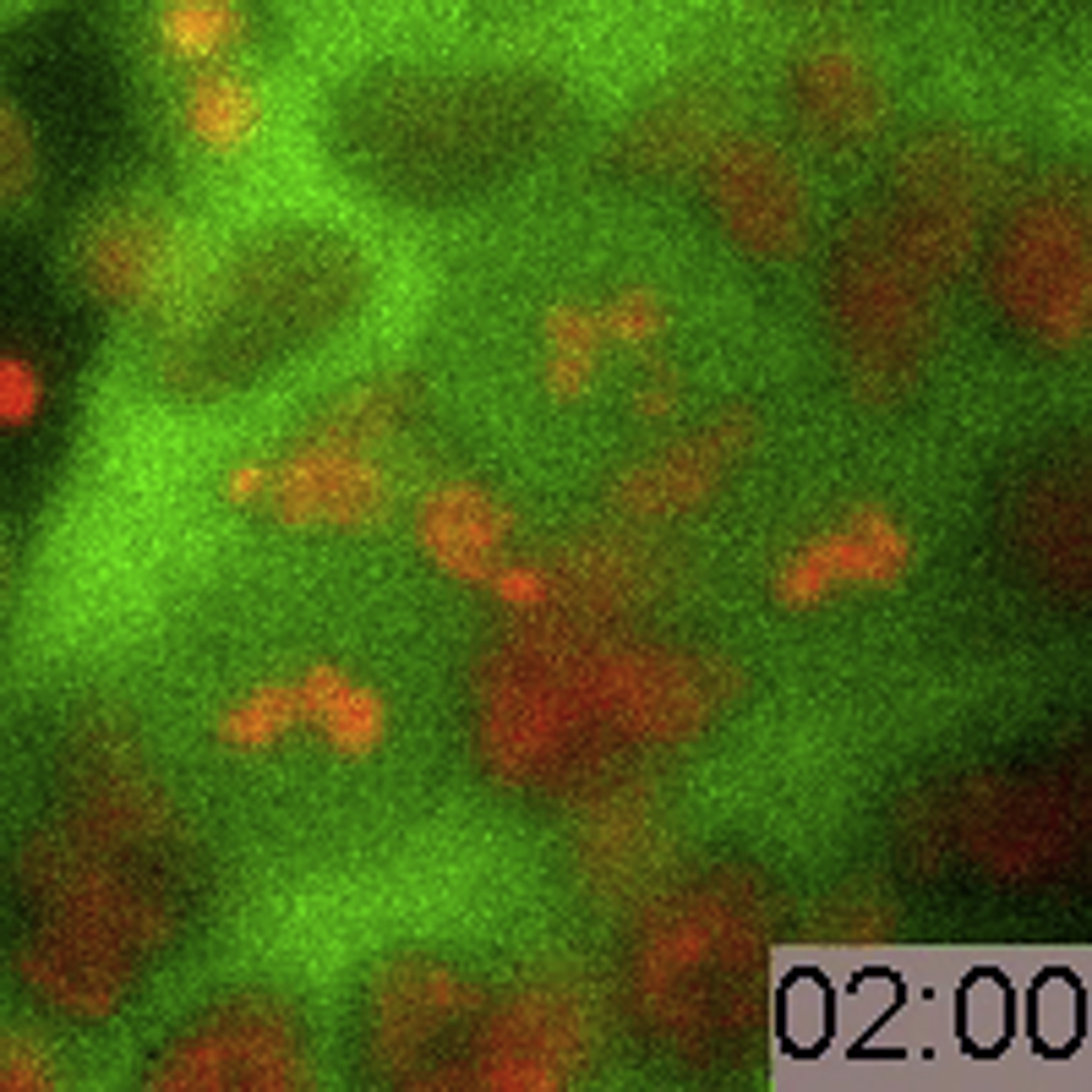

Supplement: Movie S3. Dynamic Localization of Mst-GFP and His-RFP in Larval Brain Cells — Within a cluster of larval brain cells, three are in mitosis, as assessed by chromosome condensation. The cell at the top of the field of view progresses from prometaphase to metaphase; the cell to the right is in metaphase throughout, while the cell in the bottom right undergoes anaphase. In all cells, Mst-GFP is exclusively cytoplasmic. [file mmc4.jpg]
