# Supplementary material for: Misato Controls Mitotic Microtubule Generation by Stabilizing the Tubulin Chaperone Protein-1 Complex
Source: Curr Biol. 2015 Jun 29;25(13):1777–83. doi: 10.1016/j.cub.2015.05.033 (PMC4510148; doi:10.1016/j.cub.2015.05.033)
Supplement: Document S2. Article plus Supplemental Information [file mmc5.pdf]

Report

Current Biology

Misato Controls Mitotic Microtubule Generation by Stabilizing the Tubulin Chaperone Protein-1 Complex

Highlights

- Misato interacts biochemically with the Tubulin Chaperone Protein-1 (TCP-1) complex
- Misato stabilizes the TCP-1 complex, possibly by filling its Tubulin-folding cavity
- Loss of Misato or TCP-1 complex subunits leads to similar mitotic phenotypes
- In the absence of Misato, Tubulin is unstable and unable to efficiently polymerize

Authors

Valeria Palumbo, Claudia Pellacani, Kate J. Heesom, ..., Maurizio Gatti, Silvia Bonaccorsi, James G. Wakefield

Correspondence

silvia.bonaccorsi@uniroma1.it (S.B.), j.g.wakefield@exeter.ac.uk (J.G.W.)

In Brief

Palumbo et al. show the conserved protein Misato interacts with the Tubulin Chaperone Protein-1 (TCP-1) complex in *Drosophila*. In the absence of Misato, TCP-1 subunit levels are reduced, functional Tubulin is unstable, and mitotic microtubule generation is compromised. The predicted structure of Misato suggests it acts as a molecular placeholder in the absence of Tubulin, stabilizing the TCP-1 complex.

CrossMark

Palumbo et al., 2015, Current Biology *25*, 1777–1783
June 29, 2015 ©2015 The Authors
http://dx.doi.org/10.1016/j.cub.2015.05.033

CellPress

Current Biology
Report

CellPress

Misato Controls Mitotic Microtubule Generation by Stabilizing the Tubulin Chaperone Protein-1 Complex

Valeria Palumbo,[1,2] Claudia Pellacani,[1] Kate J. Heesom,[3] Kacper B. Rogala,[4] Charlotte M. Deane,[4] Violaine Mottier-Pavie,[1,7] Maurizio Gatti,[1,5,6] Silvia Bonaccorsi,[1,*] and James G. Wakefield[2,*]
[1]Dipartimento di Biologia e Biotecnologie, Istituto Pasteur-Fondazione Cenci Bolognetti, Sapienza Università di Roma, Ple. A. Moro 5, 00185 Rome, Italy
[2]Biosciences, College of Life and Environmental Sciences, University of Exeter, Stocker Road, Exeter EX4 4QD, UK
[3]Proteomics Facility, Faculty of Medical and Veterinary Sciences, University of Bristol, Bristol BS8 1TD, UK
[4]Department of Statistics, University of Oxford, South Parks Road, Oxford OX1 3TG, UK
[5]Istituto di Biologia e Patologia Molecolari del CNR c/o Sapienza Università di Roma, 00185 Rome, Italy
[6]Institute of Molecular and Cellular Biology SD RAS, Novosibirsk 630090, Russia
[7]Present address: VIB Center for the Biology of Disease, KU Leuven Center for Human Genetics, University of Leuven, 3000 Leuven, Belgium
*Correspondence: silvia.bonaccorsi@uniroma1.it (S.B.), j.g.wakefield@exeter.ac.uk (J.G.W.)
http://dx.doi.org/10.1016/j.cub.2015.05.033
This is an open access article under the CC BY license (http://creativecommons.org/licenses/by/4.0/).

SUMMARY

Mitotic spindles are primarily composed of microtubules (MTs), generated by polymerization of α- and β-Tubulin hetero-dimers [1, 2]. Tubulins undergo a series of protein folding and post-translational modifications in order to fulfill their functions [3, 4]. Defects in Tubulin polymerization dramatically affect spindle formation and disrupt chromosome segregation. We recently described a role for the product of the conserved *misato* (*mst*) gene in regulating mitotic MT generation in flies [5], but the molecular function of Mst remains unknown. Here, we use affinity purification mass spectrometry (AP-MS) to identify interacting partners of Mst in the *Drosophila* embryo. We demonstrate that Mst associates stoichiometrically with the hetero-octameric Tubulin Chaperone Protein-1 (TCP-1) complex, with the hetero-hexameric Tubulin Prefoldin complex, and with proteins having conserved roles in generating MT-competent Tubulin. We show that RNAi-mediated in vivo depletion of any TCP-1 subunit phenocopies the effects of mutations in *mst* or the Prefoldin-encoding gene *merry-go-round* (*mgr*), leading to monopolar and disorganized mitotic spindles containing few MTs. Crucially, we demonstrate that Mst, but not Mgr, is required for TCP-1 complex stability and that both the efficiency of Tubulin polymerization and Tubulin stability are drastically compromised in *mst* mutants. Moreover, our structural bioinformatic analyses indicate that Mst resembles the three-dimensional structure of Tubulin monomers and might therefore occupy the TCP-1 complex central cavity. Collectively, our results suggest that Mst acts as a co-factor of the TCP-1 complex, playing an essential role in the Tubulin-folding processes required for proper assembly of spindle MTs.

RESULTS AND DISCUSSION

Misato Biochemically Interacts with the Tubulin Chaperone Protein-1 Complex

We previously demonstrated that mutations in *misato* (*mst*) lead to frequent monopolar spindles (~70%) with low microtubule (MT) density in *Drosophila* larval brains and that, upon MT regrowth after cold exposure, *mst* cells are primarily defective in kinetochore-driven MT generation [5].

To address the molecular function of Mst, we expressed a GFP-tagged variant in the *Drosophila* syncytial blastoderm embryo, which undergoes a series of rapid, synchronous nuclear divisions (Figure 1A; Movies S1 and S2). Expression of Mst-GFP fully rescued the lethality associated with the *mst[1]* mutation and, under the control of the V32-GAL4 female germline-specific driver, Mst-GFP was expressed in embryos at similar levels to endogenous Mst (Figure 1B). Time-lapse experiments (Figure 1A; Movies S1 and S2) showed that Mst-GFP is excluded from interphase nuclei. Upon nuclear envelope breakdown, Mst accumulates in the region encompassed by the mitotic spindle but is excluded from centrosomes and astral MTs. By anaphase, Mst-GFP is most intense in the region corresponding to the central spindle MTs. This dynamic localization was confirmed by analysis of fixed embryos immunostained for α-Tubulin and Mst (Figure S1). In contrast, the analysis of brains expressing Mst-GFP under the control of an Actin-GAL4 driver (Figure S1 and Movie S3) and immunolocalization studies on fixed larval brains [5] (Figure 2F) failed to reveal Mst enrichment on the spindle. Thus, Mst is specifically associated with spindles of syncytial embryos.

To establish the biological process in which Mst functions, we sought to identify interacting partners. Syncytial embryos, which undergo 13 mitotic divisions predominantly in the absence of zygotic transcription, contain large amounts of mitotic proteins. We developed a pipeline based on GFP-TRAP-A affinity purification

CrossMark

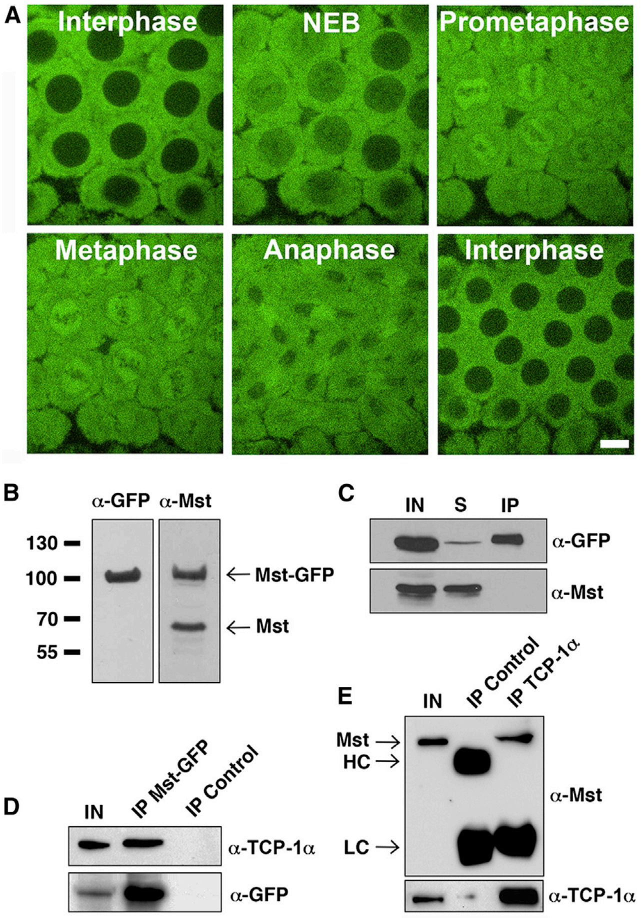

Figure 1. Mst Interacts with the TCP-1 Complex in the *Drosophila* Embryo

(A) Selected frames from a time-lapse movie of a cycle 11 syncytial embryo expressing Mst-GFP. Note that Mst accumulates on the spindle. The scale bar represents 10 μm.

(B) Western blots of extracts from Mst-GFP expressing embryos probed with anti-GFP and anti-Mst antibodies, showing that Mst-GFP and endogenous Mst are expressed at similar levels.

(C) Immunoprecipitation of Mst-GFP from embryos using a GFP-TRAP-A-based affinity purification approach; GFP-TRAP-A binds Mst-GFP, but not endogenous Mst. IN, input (Mst-GFP embryo extract) (10%); S, supernatant (extract following incubation with GFP-TRAP-A beads); IP, immunoprecipitate (GFP-TRAP-A beads, post-incubation).

(D) GFP-TRAP-A beads co-precipitate Mst-GFP and TCP-1α from embryo extracts (IP Mst-GFP). IN, input (Mst-GFP embryo extract) (10%); blocked agarose beads were used as a negative control (IP Control).

(E) Western blot showing that anti-TCP-1α antibodies precipitate Mst from wild-type embryo extracts (IP TCP1-α); IgM was used as a negative control (IP Control). IN, input (wild-type embryo extract) (10%); HC and LC, IgG heavy and light chains, respectively.

and mass spectrometry (AP-MS) combined with bioinformatics-based removal of non-specific contaminants, which allows the robust identification of GFP-bait protein interacting partners (see Supplemental Experimental Procedures). We undertook this procedure in triplicate for embryos expressing Mst-GFP. Incubation of 0–3 hr clarified embryo extracts with GFP-TRAP-A consistently depleted ~95% of Mst-GFP, without affecting levels of untagged Mst, demonstrating that Mst is a monomer in the embryo (Figure 1C). Bioinformatics-based analysis of AP precipitates identified interactors, which suggested a core functional relationship with Mst. Table 1 shows a list of Mst-GFP interacting proteins from one of these experiments, after stringent filtering (for an extended list based on less stringent filtering, see http://www.thewakefieldlab.com/ms.html). A set of eight "top hit" proteins had similar MS scores and peptide coverages as Mst itself (~1,300–3,000 and 70%–90%, respectively). These constitute all the subunits of the hetero-octameric Tubulin Chaperone Protein-1 (TCP-1) complex (Table 1). Also known as the Chaperonin Containing TCP-1 (CCT) or Tcp-1 Ring Complex (TRiC), the TCP-1 complex is an integral component of the Tubulin-folding pathway [3, 6, 7]. In this pathway, a complex termed Prefoldin [8, 9] initially interacts with newly synthesized α- and β-Tubulin, delivering them to the "donut"-shaped TCP-1 complex, which provides a suitable environment in which Tubulin can be correctly folded [3, 6–9]. A further set of conserved TCP-1 complex modulating proteins are additionally required, ensuring newly translated α- and β-Tubulin can be modified and incorporated into MTs [10]. The same pathway controls the proper folding of Actin and γ-Tubulin [11, 12].

All six subunits of the Prefoldin complex were also present in Mst-GFP AP precipitates (Table 1). Furthermore, two of the five remaining significant hits (Viaf and PDCD-5) are TCP-1 interactors. Viaf, a member of the Phosducin family, associates with the TCP-1 complex in flies [13], and its closest human homolog (PhLP-3) binds TCP-1, working antagonistically to Prefoldin [14]. Similarly, the human homolog of PDCD-5 binds to Phosducin and TCP-1β, interfering with the Tubulin-TCP-1 complex interaction [15].

The interaction between Mst and TCP-1α, the only subunit of the TCP-1 complex for which antibodies are available, was verified through reciprocal immunoprecipitation and western blotting in wild-type and Mst-GFP-expressing embryos (Figures 1D and 1E). Thus, the interaction between the TCP-1 complex and Mst reflects a normal in vivo interaction. Moreover, quantitative analysis of the MS data confirmed that similar quantities of all eight TCP-1 complex subunits and Mst were present in AP precipitates (Table 1). From this we infer that one molecule of Mst has the ability to interact with a single TCP-1 hetero-octameric complex. Notably, the Prefoldin subunits and the additional Tubulin-folding interactors were precipitated at ~10- to 100-fold-lower amounts than the TCP-1 subunits. This most likely reflects transient ternary complexes that are formed between these proteins and the TCP-1 complex [8, 9, 13–15]. In summary, we demonstrate an in vivo biochemical relationship between Mst, the TCP-1 complex, and other Tubulin-folding pathway components.

Depletion of the TCP-1 Complex Phenocopies the Loss of Mst and Merry-Go-Round

To investigate the functional relationship between Mst and its principal interactors, we compared the mitotic phenotypes elicited by depletion of either Mst, the *Drosophila* Prefoldin 3 subunit encoded by *merry-go-round* (*mgr*; [16, 17]), and the TCP-1 complex subunits. Available mutations in the genes encoding the TCP-1 complex subunits caused early embryonic lethality, preventing cytological analysis in larval brains. Similarly, in vivo

RNAi, using UAS-RNAi lines and ubiquitous drivers, produced an early lethal phenotype. We thus generated flies carrying a suitable UAS-RNAi construct and the conditional tubGAL4-tubGAL80^{ts} driver, permitting time-restricted expression of this construct.

Brain preparations from larvae grown for 72 hr at 29°C, expressing UAS-RNAi constructs against the TCP-1 complex subunits, were immunostained for Tubulin and the centrosomal marker DSpd-2 [18] and compared with *mst* and *mgr* mutant brain preparations. Consistent with previous observations, *mst* and *mgr* mutant brains displayed a metaphase arrest phenotype and frequent polyploid cells [5, 16, 17, 19]. In addition, most *mst* and *mgr* prometaphase/metaphase figures showed monopolar spindles with reduced MT density (Figures 2A and S2). Importantly, RNAi against Tcp-1α caused an ~80% reduction of the protein level and a mitotic phenotype indistinguishable from that elicited by *mst* mutations (Figures 2A–2C and S2). Moreover, RNAi against each of the other seven TCP-1 subunits also resulted in a very similar phenotype; we consistently observed high frequencies of monopolar spindles (ranging from 39.4% to 76.7%; n = 200 per each TCP-1 subunit) with strongly reduced MT density (Figures 2B, 2C, and S2).

To further investigate the functional relationship between Mst and the TCP-1 complex in spindle assembly, we analyzed MT regrowth after cold exposure in mitotic cells of TCP-1α-depleted brains. After cold-induced MT depolymerization, wild-type cells rapidly form new MTs, first from chromosomes and then from both chromosomes and centrosomes. The two MT populations merge within 5 min, giving rise to morphologically regular spindles ([5]; Figure S3). We previously showed that mutations in *mst* strongly reduce chromosome-driven MT regrowth, having little effect on MT regrowth from centrosomes [5]. Similarly, in TCP-1α-depleted brains exposed to cold and then returned at room temperature (RT), the majority of mitotic cells showed initial MT regrowth exclusively from the centrosomes; only after 10 min recovery did these cells show monopolar and bipolar spindles similar to those of untreated cells (Figure S3). Thus, the absence/reduction of Mst and TCP-1 subunits similarly affect spindle MT generation in larval neuroblasts, suggesting a functional link between Mst and the Tubulin-folding machinery.

Mst Is Required for the Stability of the TCP-1 Complex and Efficient Tubulin Polymerization

We next sought to distinguish whether Mst is a substrate for the TCP-1 and Prefoldin complexes or whether it acts as a co-factor for the Tubulin-folding pathway. We first assessed the amount of Mst, Mgr, and TCP-1α in larval brain extracts depleted of each of these proteins. In extracts from RNAi larvae depleted of any TCP-1 subunit, the level of TCP-1α was always dramatically reduced (Figure 2C). These findings indicate that all TCP-1 complex components are required for TCP-1α stability and, consistent with previous results in mammalian cells [20], suggest that all TCP-1 subunits are mutually required for complex stability. However, their reduction did not affect the levels of either Mgr or Mst (Figure 2D). Remarkably, while levels of both Mst and TCP-1α were unaffected in brain extracts from *mgr* mutants, mutations in *mst*, although not affecting Mgr, caused a strong reduction in the levels of TCP-1α (Figure 2E). These findings were corroborated by immunolocalization experiments. Similar to Mst [5], TCP-1α specifically accumulated in the cytoplasm of mitotic cells of wild-type brains but was almost undetectable in dividing cells of *mst* mutant brains (Figure 2F).

To assess whether the low level of TCP-1α observed in *mst* mutants reflects a reduction of the entire TCP-1 complex, we subjected wild-type and *mst* mutant larval extracts to size exclusion chromatography, probing separated fractions for TCP-1α. The TCP-1α of *mst* mutants, although reduced in amount, eluted at ~500–550 kD (corresponding to the predicted size of the *Drosophila* TCP-1 hetero-octameric complex) with a profile identical to wild-type TCP-1α (Figure 2G), indicating that Mst is required to stabilize the entire complex.

The TCP-1 complex in other experimental systems is essential for the correct folding of both Tubulin and Actin. However, while RNAi-mediated depletion of human TCP-1 complex subunits leads to a dramatic reduction in Tubulin levels, it has little effect on Actin [21]. To determine whether Mst depletion differentially affects Tubulin and Actin in *Drosophila*, we analyzed levels of α- and β-Tubulin, γ-Tubulin, and Actin in wild-type and *mst* mutant extracts from either brains or whole larvae. Although Tubulin levels were similar in wild-type and *mst* mutant brains, *mst* larvae displayed substantially reduced levels of α- and β-Tubulin compared to wild-type larvae (Figure 2H); this difference likely reflects variable susceptibilities of different tissues to loss of components of the Tubulin-folding pathway, as previously reported for *Drosophila* Mgr [17]. In contrast, both wild-type and *mst* mutant brains and larvae showed very similar levels of both γ-Tubulin and Actin. Moreover, size exclusion chromatography of wild-type, *mst* mutant extracts, or extracts of *Tcp1-α* RNAi larvae failed to reveal any effect on Actin level or size distribution profile, while confirming the reduction of α-Tubulin (Figure 2G).

To further investigate the specific loss and functionality of α- and β-Tubulin in *mst* mutant larvae, we performed Tubulin stability and MT sedimentation assays; we could not perform these experiments with isolated brains due to the difficulty in collecting enough tissue. First, by monitoring the amounts of Tubulin and Actin in extracts incubated at 27°C for varying times, we found that, while α-Tubulin levels remained unchanged in wild-type extracts over the course of 1 hr, α-Tubulin in *mst* mutant extracts was rapidly lost (Figure 2I). Next, we subjected extracts from wild-type, *mst*, and *Tcp1-α* RNAi larvae to in vitro MT and Actin sedimentation assays. We found that Actin polymerization was unaffected in *mst* or *Tcp1-α* RNAi extracts, although a consistent band-shift, possibly reflective of post-translational modification, was observed (Figure 2J). To control for the reduced concentration of α-Tubulin in *mst* and *Tcp1-α* RNAi larval extracts (Figure 2G), we performed the MT sedimentation assay with various diluted wild-type extracts. Even in wild-type extracts diluted 1:16, a proportion of α-Tubulin was able to pellet under polymerization conditions. In contrast, α-Tubulin in *mst* mutant larval extracts, similar to that present in *Tcp1-α* RNAi larvae, remained in the supernatant, suggesting an inability to polymerize (Figure 2K).

The Role of Mst in Spindle MT Assembly

Our data demonstrate that the functions of Mst and the TCP-1 complex are integrally linked. Mst binds the complex and is required for its stability. Moreover, Mst loss causes a mutant

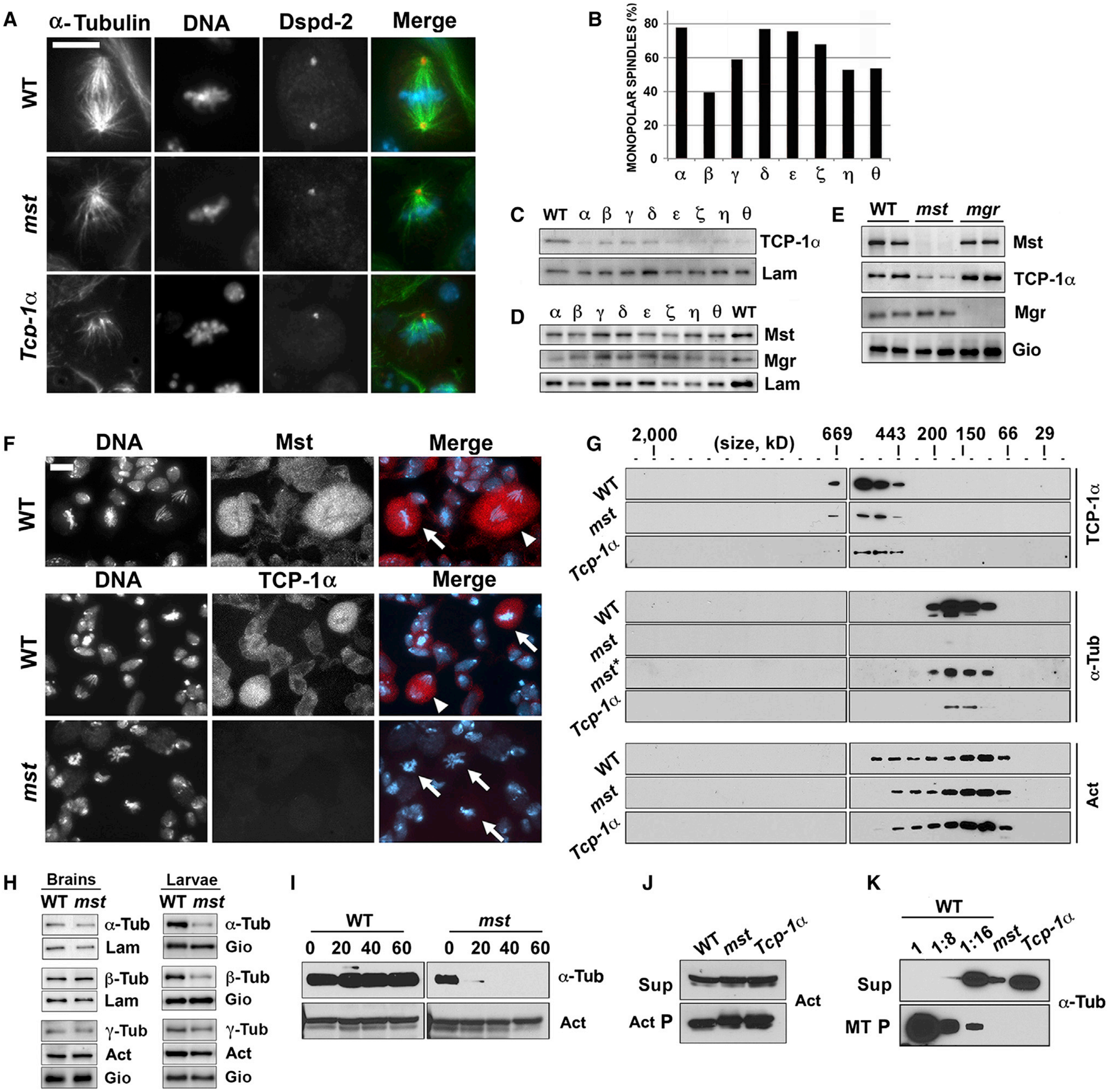

Figure 2. Mst Is Required for TCP-1 Complex Stability

(A) *mst* and TCP-1α-depleted (*Tcp-1α*) neuroblast metaphases stained for Dspd-2 (red), α-Tubulin (green), and DNA (blue) exhibit monopolar spindles with low MT density. The scale bar represents 5 μm.

(B) Frequencies of monopolar spindles observed in larval brains after RNAi-mediated depletion of TCP-1 subunits (indicated with Greek letters from α to θ).

(C) Western blots of brain extracts depleted of TCP-1 subunits probed with anti-TCP-1α and anti-Lamin (Lam, loading control) antibodies. TCP-1α is reduced in all extracts.

(D) Western blot of brain extracts depleted of TCP-1 subunits, probed for Mst, Mgr, and Lam. Mst and Mgr levels are similar to wild-type (WT).

(E) Western blot from null *mst* and *mgr* mutant brains probed for Mst, TCP-1α, Mgr, and Giotto (Gio, loading control). TCP-1α levels are strongly reduced in *mst* extracts.

(F) Localization of TCP-1α and Mst in larval brains. In WT brains, TCP-1α and Mst specifically accumulate in mitotic cells; in *mst* mutants, the TCP-1α signal is almost undetectable. Arrows and arrowheads point to metaphases and anaphases, respectively. The scale bar represents 5 μm.

(G) Western blots of larval extracts, separated using size exclusion chromatography and probed for TCP-1α, α-Tubulin (α-Tub), and Actin (Act). Mst or TCP-1α loss reduces Tubulin, but not Actin, levels. Asterisk (*) indicates overexposed WB to show Tubulin.

(H) Western blots of larval brain and whole-larvae extracts showing total levels of α- and β-Tubulin, γ-Tubulin, and Actin in WT and *mst* mutants; α- and β-Tubulin are reduced in whole larvae, but not in brains.

(legend continued on next page)

CellPress

Table 1. List of Mst-GFP Interacting Proteins

Protein Name	Percentage Coverage	Number of Peptides	MW (kDa)	Score	Mean Area
TCP1-gamma	88.97	53	59	2,924	2.47E10
TCP1-zeta	82.74	46	58	2,188	2.14E10
TCP1-alpha	79.17	41	60	2,146	1.76E10
TCP1-beta	80.93	43	58	2,017	1.58E10
TCP1-eta	84.01	45	59	1,877	2.03E10
TCP1-delta	75.80	36	57	1,818	1.55E10
Misato	74.74	33	65	1,598	2.26E10
TCP1-theta	70.33	39	59	1,367	1.93E10
TCP1-epsilon	70.85	40	59	1,345	1.18E10
Prefoldin 5	74.41	32	55	579	3.06E9
Merry-go-round (Prefoldin 3)	35.05	9	22	115	1.91E9
CG7770 (Prefoldin 6)	38.40	5	14	77	6.60E8
l(3)01239 (Prefoldin 2)	58.04	9	16	75	5.14E8
CG10635 (Prefoldin 4)	20.29	2	16	54	1.14E8
CG13993 (Prefoldin 1)	29.37	6	15	52	5.26E8
Viaf	48.33	11	27	88	2.36E8
CG8378	26.00	9	67	67	1.51E8
CHIP	37.02	9	34	65	1.43E8
PDCD-5	57.89	6	15	53	2.05E8
CG5721	28.23	10	51	51	1.38E8

Proteins identified via mass spectrometry isolated from 0–3 hr Mst-GFP-expressing *Drosophila* embryo extracts after stringent filtering (see Supplemental Experimental Procedures). The proteins shown have MS scores of >50 and coverage percentages of >20%, respectively. Mean area corresponds to Top 3 Protein Quantification (T3PQ), the mean of the three highest abundance peptides identified for each protein. Mst is identified with a similar score and mean area to all eight subunits of the TCP-1 complex. All subunits of the Prefoldin complex are also co-precipitated, albeit at approximately 10-fold-lower abundance. Similar profiles were obtained for the TCP-1 subunits in the other two experiments.

phenotype indistinguishable from that elicited by loss of any TCP-1 subunit, and the biochemical properties of extracts depleted of either Mst or TCP1-α are identical. Together, these results indicate that Mst directly regulates TCP-1 complex structure and function. Mst was initially described as having primary structural motifs similar to those found in the Tubulin superfamily members [19, 22]. We therefore sought to determine whether Mst could be structurally related to Tubulins at the tertiary level. To do this, we composed a three-dimensional model of *Drosophila* Mst. Briefly, we identified sequences homologous to Mst in the α-, β-, and γ-Tubulin and FtsZ family of proteins, structurally aligned the available homologous structures, and merged Mst to this alignment. This final alignment was then used for comparative modeling using the Rosetta software suite (Figures 3 and S4; Supplemental Experimental Procedures).

The general secondary and tertiary structural elements found within both Tubulins and FtsZ map coherently onto Mst, strongly supporting the notion that Mst is a distant member of the Tubulin superfamily (Figure 3A). However, Mst contains additional stretches of amino acids that are predicted to be structurally disordered and that possibly affect both the nucleotide binding and oligomerization activities elicited by Tubulins (Figure 3A and S4). Upon superimposition of the Mst model onto the model of bovine Tubulin:TCP-1 complex [23], we found that Mst is capable of filling the internal cavity of the TCP-1 complex (Figure 3B). Although its additional loops exceed the cavity boundary, we hypothesize that the flexibility of these disordered protein stretches allows additional interactions with the TCP-1 complex. Therefore, although further work will be required to determine whether Mst does indeed sit within the TCP-1 complex cavity in vivo, our modeling is consistent with a scenario in which Mst stabilizes the TCP-1 complex in the absence of a substrate, through a Tubulin-like interaction.

Our study also highlights the existence of tissue-specific requirements for Tubulin folding and MT polymerization. We have shown that Mst associates with embryonic spindles, consistent with the report that *Drosophila* TCP-1 subunits co-sediment with MTs from early embryos [24]. However, Mst does not appear to be enriched in larval brain spindles ([5]; Figures 2F and S1). A possible explanation for this difference is that the rapid assembly of syncytial embryonic spindles [25] requires a high local concentration of assembly-competent Tubulin. A spindle-anchored folding machinery would, however, not be necessary in cells surrounded by a plasma membrane (such as brain cells), where sufficient folded Tubulin could be provided by increasing the intracellular concentration of the folding complexes in anticipation of mitosis.

Finally, the in vivo MT regrowth experiments presented here and previously [5] demonstrate that, in either *mst* mutant or *Tcp-1* RNAi background, centrosome-driven MT regrowth after cold treatment is less affected than chromosome-/kinetochore-induced regrowth. The simplest explanation is that, following cold-induced depolymerization, kinetochores and centrosomes nucleate MTs with different dynamics. Cold treatment removes the vast majority of cellular MTs, probably leaving only short, cold-resistant MT seeds at centrosomes. While rewarming of wild-type cells essentially "reboots" spindle assembly pathways, allowing both polymerization from centrosomes and ab-initio nucleation/polymerization from kinetochores, the reduced pool of assembly-competent Tubulin present in *mst* or *Tcp-1* mutant cells would be preferentially incorporated into the existing MT stubs at the centrosomes. An alternative hypothesis may reflect that the chromosome- and centrosome-driven MT formation pathways governing *Drosophila* cell division are at least in part under separate genetic control [5, 25, 26].

In summary, our study identifies Mst as a factor required for the stability of the TCP-1 complex, ultimately controlling the

(I) Western blots of WT or *mst* larval extracts incubated at 27°C for 20, 40, or 60 min, probed for α-Tubulin and Actin. Tubulin is rapidly lost in *mst* extracts.
(J) Western blot of an Actin sedimentation assay performed with WT, *mst*, and *Tcp-1α* RNAi larval extracts. Actin is able to polymerize in all extracts.
(K) Western blot of a MT sedimentation assay. WT extracts were diluted 1:8 and 1:16 to control for reduced Tubulin levels in *mst* and *Tcp-1α* RNAi extracts. Tubulin is competent to polymerize in WT, but not in *mst* or *Tcp-1α* RNAi extracts.

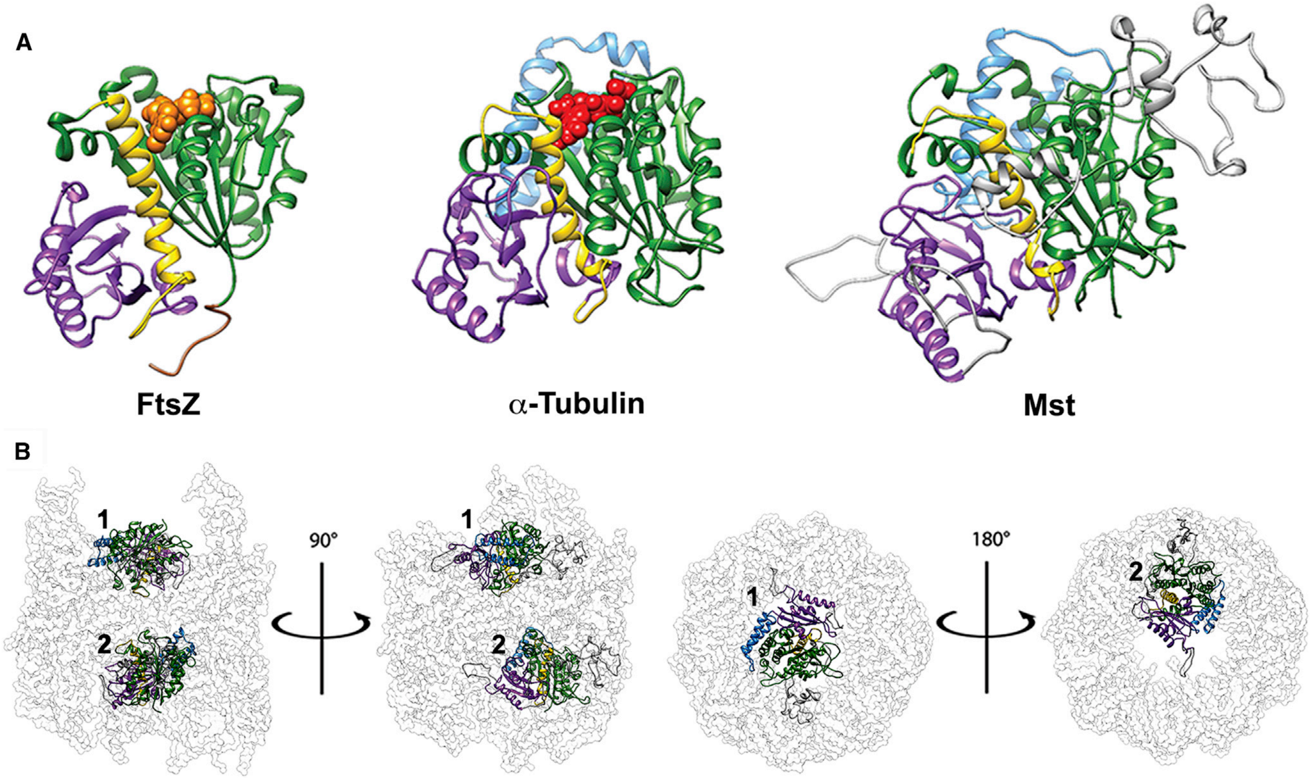

Figure 3. Structural Comparison of FtsZ, Tubulin, and Mst and Model of Mst in the TCP-1 Complex

(A) Three-dimensional models of prokaryotic FtsZ (2VAW A), eukaryotic α-Tubulin (4I4T A), and Mst (Rosetta), annotated with Tubulin/FtsZ structural elements; bound nucleotides are drawn as spheres. Green indicates GTPase domain; yellow indicates helix 7; purple indicates activation domain; blue indicates C-terminal extension; orange indicates GDP; red indicates GTP; and gray indicates Mst loops.

(B) Superimposition of Mst onto a partial model of Tubulin within the bovine TCP-1 complex (PDB: 2XSM). TCP-1 complex is represented as surface (white) and Mst as cartoons, colored as in (A). Top panels are front views of the complex, with two superimposed molecules of Mst (1 and 2) related by a 90° rotation around the depicted axis. Bottom panels are above and below views of the complex, respectively, showing only one superimposed Mst model (1 or 2), closer to the viewer, related by a 180° rotation around the depicted axis.

stability and polymerizing competency of α- and β-Tubulin within the fly. Mst is a conserved protein; future work will clarify whether it is a TCP-1 complex cofactor with a mitotic role in humans.

SUPPLEMENTAL INFORMATION

Supplemental Information includes Supplemental Experimental Procedures, four figures, and three movies and can be found with this article online at http://dx.doi.org/10.1016/j.cub.2015.05.033.

AUTHOR CONTRIBUTIONS

V.P. performed most of the experiments. C.P. and K.J.H. performed the biochemical and MS analyses, respectively. V.M.-P. generated Mst-GFP. Modeling of Mst was undertaken by K.B.R. under the supervision of C.M.D. J.G.W. performed the size exclusion chromatography. V.P., S.B., M.G., and J.G.W. conceived the experiments, analyzed the data, and wrote the paper.

ACKNOWLEDGMENTS

We would like to thank Elisabetta Bucciarelli, Pete Jones, Silvana Caristi, Mirella Vivoli, James Marks, and Emily Richardson for help and advice and Guillermo Montoya for TCP-1 and Tubulin structural data. V.P. was funded by a PRIN grant from MIUR to S.B. and by the BBSRC grant BB/K017837/1 to J.G.W. M.G. was supported in part by a grant from the Ministry of Education and Science of Russian Federation (14.Z50.31.0005).

Received: November 26, 2014
Revised: April 14, 2015
Accepted: May 15, 2015
Published: June 18, 2015

REFERENCES

1. Weisenberg, R.C. (1972). Microtubule formation in vitro in solutions containing low calcium concentrations. Science *177*, 1104–1105.
2. Walczak, C.E., and Heald, R. (2008). Mechanisms of mitotic spindle assembly and function. Int. Rev. Cytol. *265*, 111–158.
3. Hartl, F.U., and Hayer-Hartl, M. (2002). Molecular chaperones in the cytosol: from nascent chain to folded protein. Science *295*, 1852–1858.
4. Westermann, S., and Weber, K. (2003). Post-translational modifications regulate microtubule function. Nat. Rev. Mol. Cell Biol. *4*, 938–947.
5. Mottier-Pavie, V., Cenci, G., Vernì, F., Gatti, M., and Bonaccorsi, S. (2011). Phenotypic analysis of misato function reveals roles of noncentrosomal microtubules in Drosophila spindle formation. J. Cell Sci. *124*, 706–717.
6. Yaffe, M.B., Farr, G.W., Miklos, D., Horwich, A.L., Sternlicht, M.L., and Sternlicht, H. (1992). TCP1 complex is a molecular chaperone in tubulin biogenesis. Nature *358*, 245–248.
7. Yébenes, H., Mesa, P., Muñoz, I.G., Montoya, G., and Valpuesta, J.M. (2011). Chaperonins: two rings for folding. Trends Biochem. Sci. *36*, 424–432.

8. Vainberg, I.E., Lewis, S.A., Rommelaere, H., Ampe, C., Vandekerckhove, J., Klein, H.L., and Cowan, N.J. (1998). Prefoldin, a chaperone that delivers unfolded proteins to cytosolic chaperonin. Cell *93*, 863–873.

9. Siegert, R., Leroux, M.R., Scheufler, C., Hartl, F.U., and Moarefi, I. (2000). Structure of the molecular chaperone prefoldin: unique interaction of multiple coiled coil tentacles with unfolded proteins. Cell *103*, 621–632.

10. Willardson, B.M., and Howlett, A.C. (2007). Function of phosducin-like proteins in G protein signaling and chaperone-assisted protein folding. Cell. Signal. *19*, 2417–2427.

11. Gao, Y., Thomas, J.O., Chow, R.L., Lee, G.H., and Cowan, N.J. (1992). A cytoplasmic chaperonin that catalyzes β-actin folding. Cell *69*, 1043–1050.

12. Melki, R., Vainberg, I.E., Chow, R.L., and Cowan, N.J. (1993). Chaperonin-mediated folding of vertebrate actin-related protein and gamma-tubulin. J. Cell Biol. *122*, 1301–1310.

13. Guruharsha, K.G., Rual, J.F., Zhai, B., Mintseris, J., Vaidya, P., Vaidya, N., Beekman, C., Wong, C., Rhee, D.Y., Cenaj, O., et al. (2011). A protein complex network of Drosophila melanogaster. Cell *147*, 690–703.

14. Stirling, P.C., Srayko, M., Takhar, K.S., Pozniakovsky, A., Hyman, A.A., and Leroux, M.R. (2007). Functional interaction between phosducin-like protein 2 and cytosolic chaperonin is essential for cytoskeletal protein function and cell cycle progression. Mol. Biol. Cell *18*, 2336–2345.

15. Tracy, C.M., Gray, A.J., Cuéllar, J., Shaw, T.S., Howlett, A.C., Taylor, R.M., Prince, J.T., Ahn, N.G., Valpuesta, J.M., and Willardson, B.M. (2014). Programmed cell death protein 5 interacts with the cytosolic chaperonin containing tailless complex polypeptide 1 (CCT) to regulate β-tubulin folding. J. Biol. Chem. *289*, 4490–4502.

16. Gonzalez, C., Sunkel, C.E., and Glover, D.M. (1998). Interactions between mgr, asp, and polo: asp function modulated by polo and needed to maintain the poles of monopolar and bipolar spindles. Chromosoma *107*, 452–460.

17. Delgehyr, N., Wieland, U., Rangone, H., Pinson, X., Mao, G., Dzhindzhev, N.S., McLean, D., Riparbelli, M.G., Llamazares, S., Callaini, G., et al. (2012). Drosophila Mgr, a Prefoldin subunit cooperating with von Hippel Lindau to regulate tubulin stability. Proc. Natl. Acad. Sci. USA *109*, 5729–5734.

18. Giansanti, M.G., Bucciarelli, E., Bonaccorsi, S., and Gatti, M. (2008). Drosophila SPD-2 is an essential centriole component required for PCM recruitment and astral-microtubule nucleation. Curr. Biol. *18*, 303–309.

19. Miklos, G.L., Yamamoto, M., Burns, R.G., and Maleszka, R. (1997). An essential cell division gene of Drosophila, absent from Saccharomyces, encodes an unusual protein with tubulin-like and myosin-like peptide motifs. Proc. Natl. Acad. Sci. USA *94*, 5189–5194.

20. Kunisawa, J., and Shastri, N. (2003). The group II chaperonin TRiC protects proteolytic intermediates from degradation in the MHC class I antigen processing pathway. Mol. Cell *12*, 565–576.

21. Grantham, J., Brackley, K.I., and Willison, K.R. (2006). Substantial CCT activity is required for cell cycle progression and cytoskeletal organization in mammalian cells. Exp. Cell Res. *312*, 2309–2324.

22. Gurvitz, A., Hartig, A., Ruis, H., Hamilton, B., and de Couet, H.G. (2002). Preliminary characterisation of DML1, an essential Saccharomyces cerevisiae gene related to misato of Drosophila melanogaster. FEMS Yeast Res. *2*, 123–135.

23. Muñoz, I.G., Yébenes, H., Zhou, M., Mesa, P., Serna, M., Park, A.Y., Bragado-Nilsson, E., Beloso, A., de Cárcer, G., Malumbres, M., et al. (2011). Crystal structure of the open conformation of the mammalian chaperonin CCT in complex with tubulin. Nat. Struct. Mol. Biol. *18*, 14–19.

24. Hughes, J.R., Meireles, A.M., Fisher, K.H., Garcia, A., Antrobus, P.R., Wainman, A., Zitzmann, N., Deane, C., Ohkura, H., and Wakefield, J.G. (2008). A microtubule interactome: complexes with roles in cell cycle and mitosis. PLoS Biol. *6*, e98.

25. Hayward, D., Metz, J., Pellacani, C., and Wakefield, J.G. (2014). Synergy between multiple microtubule-generating pathways confers robustness to centrosome-driven mitotic spindle formation. Dev. Cell *28*, 81–93.

26. Bucciarelli, E., Pellacani, C., Naim, V., Palena, A., Gatti, M., and Somma, M.P. (2009). Drosophila Dgt6 interacts with Ndc80, Msps/XMAP215, and gamma-tubulin to promote kinetochore-driven MT formation. Curr. Biol. *19*, 1839–1845.

Current Biology

Supplemental Information

Misato Controls Mitotic Microtubule Generation by Stabilizing the Tubulin Chaperone Protein-1 Complex

Valeria Palumbo, Claudia Pellacani, Kate J. Heesom, Kacper B. Rogala, Charlotte M. Deane, Violaine Mottier-Pavie, Maurizio Gatti, Silvia Bonaccorsi, and James G. Wakefield

Supplementary Figure S1

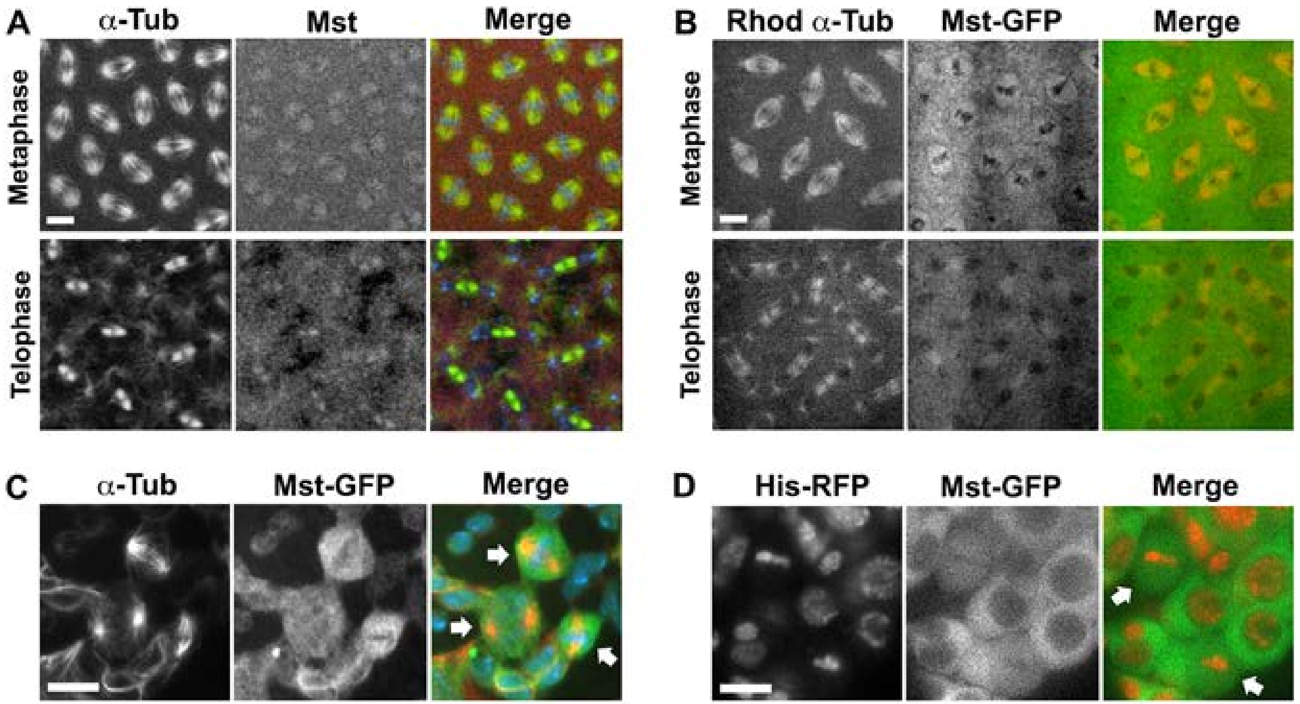

Supplementary Figure S1. Mst localisation in living and fixed embryos and brain cells. (A) Metaphase and telophase figures from cycle 12 fixed embryos stained for α-Tubulin (green), Mst (red) and DNA (blue). (B) Selected frames from a time-lapse movie of a cycle 11 embryo expressing Mst-GFP (green) and injected with Rhodamine-labelled α-Tubulin (Rhod α-Tub, red). In both live and fixed embryos, Mst co-localises with the region of the mitotic spindle, but not with centrosomes, during metaphase, and with the region of the central spindle during telophase. (C) Brain squashes from Mst-GFP expressing larvae immunostained for GFP and Tubulin. In merged figures, α-Tubulin is red, Mst-GFP is green and DNA is blue. (D) Single frame from a live preparation of larval neuroblasts expressing the red fluorescent protein-labeled histone H2AvD (His-RFP, red) and Mst-GFP (green). In both live and fixed brain cells, Mst-GFP is cytoplasmic during mitosis. Scale bar in A and B, 10 μm; in C and D, 5 μm. Related to Figure 1.

Supplementary Figure S2

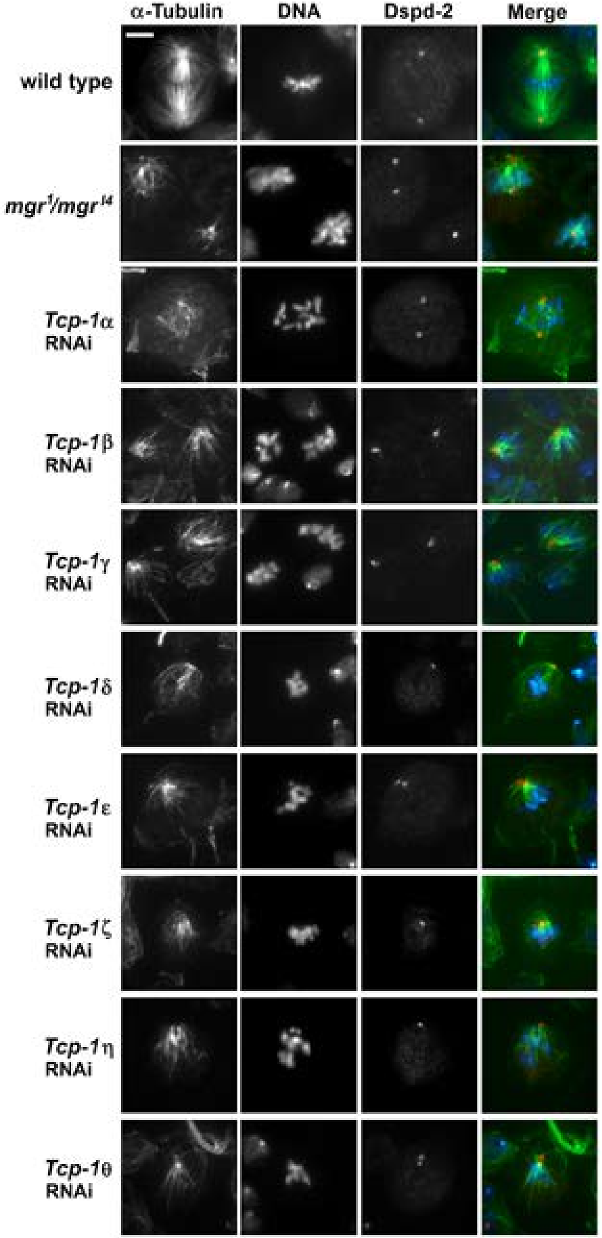

Supplementary Figure S2. *mgr* mutant and TCP-1 complex subunit RNAi phenotypes
Examples of mitotic spindles observed in brains from mgr^1/mgr^{i4} mutant larvae, and larvae expressing RNAi constructs against the indicated TCP-1 complex subunits. In merged figures, DNA is blue, α-Tubulin green, and DSpd-2 red. Scale bar, 5 μm. Related to Figure 2.

Supplementary Figure S3

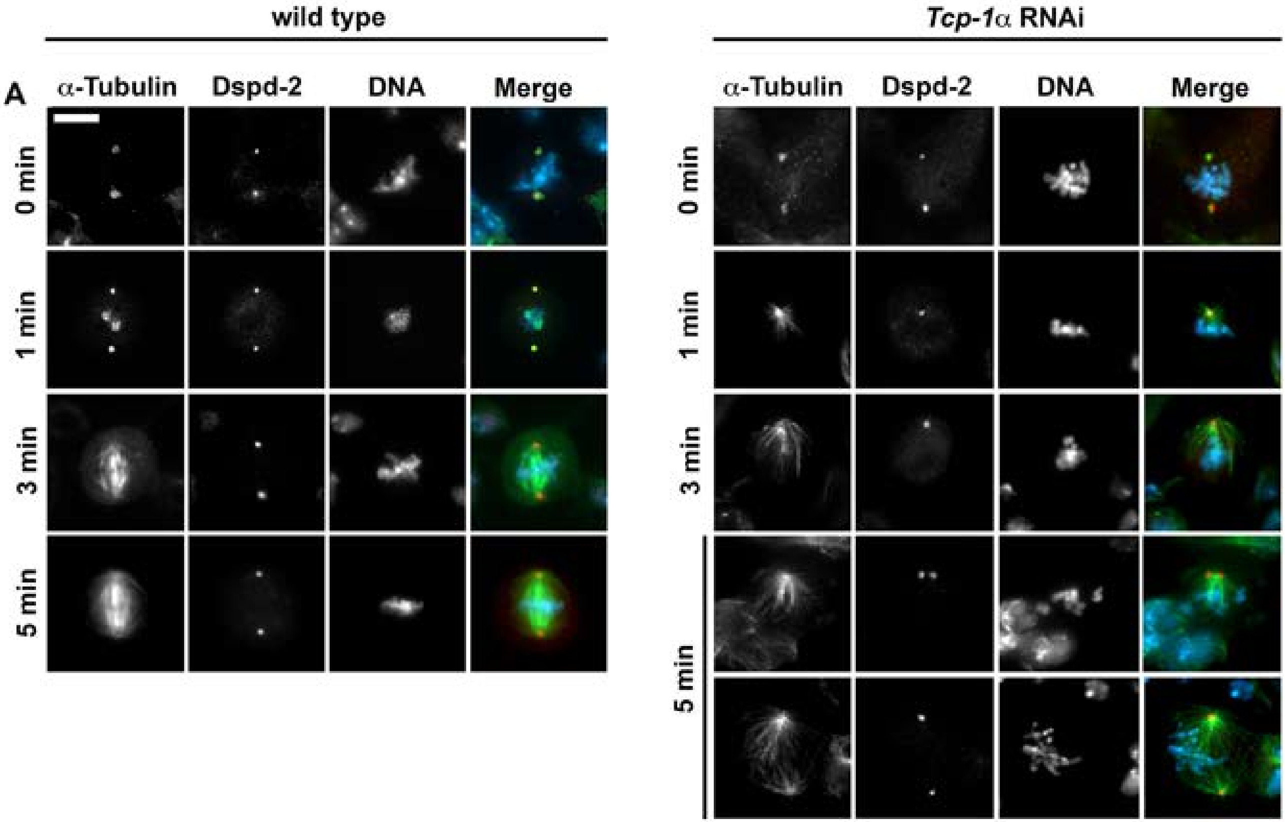

B

Genotype, time	# of cells	ch %	cn %	ch, cn %
wt, 1 min	90	62	2	36
wt, 3 min	70	40	0	60
wt, 5 min	75	0	0	100

C

Genotype, time	# of cells	ch %	cn %	ch, cn %
Tcp-1α RNAi, 1 min	60	8	80	12
Tcp-1α RNAi, 3 min	66	7	72	21
Tcp-1α RNAi, 5 min	50	6	66	28

Supplementary Figure S3. Microtubule regrowth after cold-induced depolymerisation
(A) After 50 min of cold treatment, MTs are completely depolymerized in both wild type and TCP1α-depleted brains. After 1 and 3 min recovery at room temperature, in wild type metaphase cells, microtubule (MT) regrowth occurs mainly near the chromosomes or from both the chromosomes and the centrosomes; after 5 min, most spindles are fully assembled. In TCP-1α-depleted cells, MT regrowth near the chromosomes is dramatically reduced at any time, and after 5 min most spindles only exhibit abnormally long centrosome-nucleated MTs. Cells were stained for DSpd-2 (red), Tubulin (green) and DNA (blue). Scale bar, 5 μm. (B, C) Frequencies of prometaphases/metaphases (P/M) from wild type (B) and TCP-1α-depleted brains (C) showing MT nucleation from the chromosomes only (ch), the centrosomes only (cn), or from both the chromosomes and the centrosomes (ch, cn). Related to Figure 2.

Supplementary Figure S4

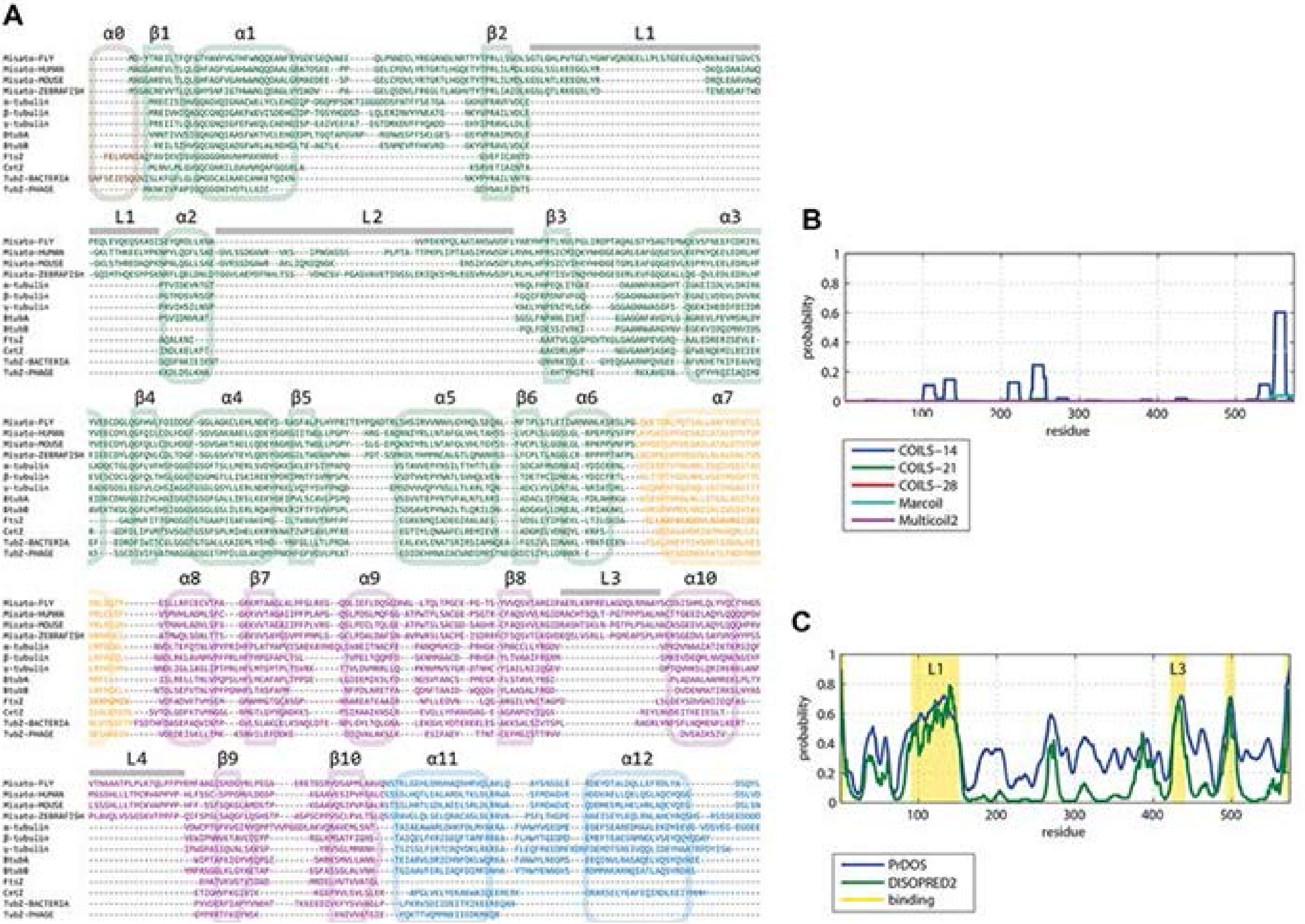

Supplementary Figure S4. Structural Bioinformatics Analysis of Misato. (A) Multiple sequence alignment of Mst and tubulin/FtsZ group of proteins. MSA7 was used for comparative modeling with RosettaCM (only representative sequences are shown; see Supplementary Experimental Procedures for the complete list), constructed by merging a structural alignment of Tubulin/FtsZ (MSA4) and the close homology alignment of Mst proteins (MSA6). Sequences used: Mst-FLY (RefSeq: NP_523435.1), Mst-HUMAN (RefSeq: NP_060586.2), Mst-MOUSE (GenBank: AAH08103.1), Mst-ZEBRAFISH (RefSeq: NP_956181.1), α-tubulin (PDB: 4I4T A), β-tubulin (PDB: 4I4T B), γ-tubulin (PDB: 3CB2 A), BtubA (PDB: 2BTO A), BtubB (PDB: 2BTQ B), FtsZ (PDB: 2VAW A), CetZ (PDB: 3ZID A), TubZ-BACTERIA (PDB: 4EI7 A), TubZ-PHAGE (PDB: 3V3T A). Colouring scheme: brown, N-terminal extensions; green, GTPase domain; yellow, helix 7; purple, activation domain; blue, C-terminal extension. Secondary structural elements are highlighted by corresponding boxes, with additional Mst loops marked by horizontal gray bars. (B) Coiled-coil formation propensity of Mst, predicted by COILS (window-14, blue; window-21, green; window-28, red; MTK matrix), Marcoil (cyan; MTK matrix), and Multicoil2 (magenta). (C) Disorder tendency predicted by PrDOS (blue) and DISOPRED2 (green). Yellow, transparent boxes signify potential binding sites in the disordered regions, some of which comprise additional loops of Mst: L1 and L3. Related to Figure 3.

SUPPLEMENTARY EXPERIMENTAL PROCEDURES

Drosophila strains

The inducible *mst*-GFP fly strain (carrying a pUASp-Mst-GFP element) was generated by cloning the full-length *mst* gene into the pPWG vector via pENTR/D-TOPO (Invitrogen). The plasmid was injected into *w1118* embryos by Bestgene Inc (Chino Hills, California), using standard procedures. Expression in the female germline was driven by V32-GAL4 (kindly provided by M. Bettencourt-Dias). For *in vivo* time-lapse imaging in larval brains, a strain was created containing both a P{H2AvD-RFP} insertion that constitutively expresses the red fluorescent protein-labelled histone H2AvD and the Mst-GFP transgene induced by the P{Act5C-GAL4}25FO1 driver insertion (FlyBase). The mst^{C27} and mst^{LB20} mutant alleles [S1] were obtained from Bloomington Stock Center. The *merry-go-round* alleles mgr^{1} and mgr^{J4} [S2, S3] were used to construct a late lethal *mgr* mutant genotype. TCP-1 complex subunits (TCP-1α-θ) RNAi fly strains (v34070, v108615, v106093, v106099, v109505, v109734, v 108585, v103905) were all obtained from the Vienna *Drosophila* RNAi center [S4]. The silencing was achieved by combining a single copy of *UAS-RNAi* transgene with a single copy of tub-GAL4, tub-GAL80^{ts} (kindly provided by T. Megraw; [S5]); cytological analysis was performed after 72 h of temperature shift from GAL4-suppressive (18°C) to permissive (29°C) temperature. Oregon-R strain was used as wild type. All flies were reared according to standard procedures and maintained at 25°C (unless otherwise specified). The genetic markers and special chromosomes are described in detail in FlyBase (http://www.flybase.org).

***In vivo* imaging**

For *in vivo* time-lapse imaging of embryos, dechorionated 1-2 h old embryos expressing GFP-Mst were aligned in heptane glue on 22 × 50 mm coverslips, and covered with a 1:1 mixture of Halocarbon oil 700 and Halocarbon oil 27 (Sigma). For co-imaging of microtubules, Mst-GFP expressing embryos were injected with X-Rhodamine labelled tubulin (Cytoskeleton Inc.) at 5mg/ml in injection buffer (50mM K-HEPES pH 7.4, 50mM KCl) using an Eppendorf Inject Man NI 2 and Femtotips® II needles (Eppendorf). Imaging was performed using a Visitron Systems Olympus IX81 microscope equipped with a CSO-X1 spinning disk using a UPlanS APO 1.3 NA (Olympus) 60X objective. Five 1-µm slice stacks were acquired at a 400 ms exposure per slice, at a constant room temperature of 22°C. Image processing and analysis was performed using ImageJ to produce accumulated projections of the two 1-µm focal planes containing the highest signal intensity.

In vivo imaging of larval brains was carried out as described [S6]. Cells were examined using a calibrated Prior Proscan stepping motor, with an EM-CCD camera (Cascade II, Photometrics) connected to a spinning-disk confocal head (CarvII, Beckton Dickinson) mounted on an inverted microscope (Eclipse TE2000S, Nikon). The objective used was 100X (NA = 1.3). Image acquisition was controlled through the Metamorph software package (Universal Imaging, Downing Town, PA). Images were collected at 1-min intervals, and 7 fluorescence optical sections were captured at 1-µm z steps. Movies were created with Metamorph software; each fluorescent image shown is the maximum-intensity projection of all the sections.

Western blotting

Samples were run on standard SDS-PAGE gels, blotted and incubated with the following primary antibodies: mouse anti-Mst (1:5,000; Santa Cruz Biotech), mouse anti-α-Tubulin (1:10,000; Sigma, DM1A clone); rabbit anti-β-Tubulin (1:5,000; DSHB, E7 clone); rabbit anti-γ-Tubulin (1:2,000; Sigma, QG-17), mouse anti-GFP (1:10,000; Roche); mouse anti-Actin antibody (Sigma, AC-40 clone); rat anti-TCP-1α (1:1,000; Abcam); rabbit anti-Mgr (1:1,000; gift of D. Glover); mouse anti-Lamin Dm0 (1:5,000; DSHB, ADL101 clone); rabbit anti-Giotto (1:4,000; [S7]). For detection, the following HRP conjugated secondary antibodies were used: anti-mouse IgG (Sigma); anti-rabbit IgG (GE Healthcare), anti-rat IgG (GE Healthcare), all diluted 1:5-10,000. Samples were visualised by using enzyme-linked chemiluminescence and X-ray film, or imaged using ECL detection kit (GE Healthcare). Band intensities were quantified by densitometric analysis with Image Lab software (Bio-Rad).

GFP-TRAP-A isolation of Mst and immunoprecipitation

Flies expressing full length Mst-GFP under the control of an inducible promoter were crossed with the *V32-GAL4*/CyO strain. Batches of 0-3 h old embryos laid by cages of 1-10 day-old flies were dechorionated, weighted, flash frozen in N_2 (l) and stored at -80°C. For MS analysis, the following procedure was undertaken on three separate occasions: ~0.4 g of frozen embryos were homogenized in 1.5 ml of C buffer (50 mM HEPES [pH 7.4], 50 mM KCl, 1 mM $MgCl_2$, 1 mM EGTA, 0.1% IGEPAL CA-630, protease inhibitors (Roche)). Extract was clarified through centrifugation at 10,000 g for

10 min, 100,000 g for 30 min, and 100,000 g for a further 10 min. Clarified extract was incubated with 15 µl GFP-TRAP-A beads or blocked agarose beads (bab-20) (Chromotek) equilibrated in C Buffer for 2 h at 4°C. Mst-GFP/GFP-TRAP-A beads were then washed 4 times with ice-cold C buffer and stored at -20°C. For standard immunoprecipitations, between 0.1-0.2 g of frozen embryos were homogenized in proportional quantities of C buffer, clarified as above, and incubated with 30 μl of equilibrated Protein G sepharose beads (GE Healthcare) conjugated to anti-TCP-1α antibody for 2 h at 4°C , prior to extensive washes. Non-specific rat IgGs (Sigma) were used as co-IP negative controls.

Mass spectrometric analysis

Samples were run ~1 cm into the separating region of an SDS-PA gel, cut as a single slice and subjected to in-gel tryptic digestion using a DigestPro automated digestion unit (Intavis Ltd.). The resulting peptides were fractionated using a Dionex Ultimate 3000 nanoHPLC system in line with an LTQ-Orbitrap Velos mass spectrometer (Thermo Scientific). In brief, peptides in 1% (vol/vol) formic acid were injected onto an Acclaim PepMap C18 nano-trap column (Dionex). After washing with 0.5% (vol/vol) acetonitrile 0.1% (vol/vol) formic acid peptides were resolved on a 250 mm × 75 µm Acclaim PepMap C18 reverse phase analytical column (Dionex) over a 150 min organic gradient, using 7 gradient segments (1-6% solvent B over 1 min, 6-15% B over 58 min, 15-32% B over 58 min, 32-40% B over 3 min, 40-90% B over 1 min, held at 90% B for 6 min and then reduced to 1% B over 1 min) with a flow rate of 300 nl min^{-1}. Solvent A was 0.1% formic acid and Solvent B

was aqueous 80% acetonitrile in 0.1% formic acid. Peptides were ionized by nano-electrospray ionization at 2.1 kV using a stainless steel emitter with an internal diameter of 30 μm (Thermo Scientific) and a capillary temperature of 250°C. Tandem mass spectra were acquired using an LTQ- Orbitrap Velos mass spectrometer controlled by Xcalibur 2.1 software (Thermo Scientific) and operated in data-dependent acquisition mode. The Orbitrap was set to analyze the survey scans at 60,000 resolution (at m/z 400) in the mass range m/z 300 to 2000 and the top twenty multiply charged ions in each duty cycle selected for MS/MS in the LTQ linear ion trap. Charge state filtering, where unassigned precursor ions were not selected for fragmentation, and dynamic exclusion (repeat count, 1; repeat duration, 30 s; exclusion list size, 500) was used. Fragmentation conditions in the LTQ were as follows: normalized collision energy, 40%; activation q, 0.25; activation time, 10 ms; and minimum ion selection intensity, 500 counts.

The raw data files were processed and quantified using Proteome Discoverer software v1.2 (Thermo Scientific) and searched against the dmel-all-translation-r5.47 database using the SEQUEST (Ver. 28 Rev. 13) algorithm. Peptide precursor mass tolerance was set at 10ppm, and MS/MS tolerance was set at 0.8Da. Search criteria included carbamidomethylation of cysteine (+57.0214) as a fixed modification and oxidation of methionine (+15.9949) as a variable modification. Searches were performed with full tryptic digestion and a maximum of 1 missed cleavage was allowed. The reverse database search option was enabled and all peptide data was filtered to satisfy false discovery rate (FDR) of 5%.

Bioinformatics filtering of MS data

For stringent filtering, MS results were filtered by removing protein IDs with (i) single peptide hits, (ii) <20% peptide:protein coverage and (iii) overall MS Scores of <50. Through our on-going studies, we have produced a database of MS data accumulated from eight independent control GFP-TRAP-A experiments, each using extracts from ~0.4 g 0-3 h embryos expressing GFP-fusions to proteins in which a bait protein was not precipitated (i.e. negative controls). A spread-sheet incorporating these data was used to produce a list of false-positive protein IDs and their associated highest overall MS Score. Filtered Mst-GFP AP-MS results were cross-referenced against this database. Any protein ID that was either not identified in negative control list or was identified in negative controls with MS Scores of at least 4 fold less than in Mst-GFP was kept, while all other protein IDs were discarded. Each of the three replicate experiments produced similar results, with all TCP-1 complex subunits identified with high confidence in each case. The result of this stringent combined filtering/false-positive for one of these datasets is shown in Table 1. A less stringent filtering of this dataset (discarding only <10% peptide:protein coverage and (iii) overall MS Scores of <30) can be found on the Wakefield lab web-site (www.thewakefieldlab.com/ms.html), demonstrating the validity of the stringent analysis in maximising the confidence of the interaction dataset.

Cytology

For immunofluorescence experiments, 1-2 h old embryos were collected at 25ºC on agar plates and dechorionated in 50% bleach. After removal of the

vitelline membrane in a mixture of methanol and heptane (1:1), embryos were fixed for 30 min in 3.7% formaldehyde in PBS under gentle agitation at room temperature and blocked for 1 hour in 0.3% Triton X-100 PBS with 3% BSA before staining. For double immuno-staining of Mst and spindle MTs, embryos were first incubated O/N at 4°C with a monoclonal anti-Mst antibody (1:50; Santa Cruz Biotech), then for 1 hour at room temperature with Rhodamine-conjugated anti-mouse IgG (1:50; Jackson Laboratories) and finally with a FITC-conjugated anti α-Tubulin antibody (1:150, Sigma) for 2 hours at room temperature. Preparations were stained with TOTO-3 DNA dye (1:1,000; Life Technologies) for 10 min at room temperature and then mounted in Vectashield medium H-1000 (Vector Laboratories). Confocal analysis was performed with a laser scanning inverted microscope Zeiss LSM 780 (Zeiss, Oberckochen, Germany) equipped with a 63X/1.4 Oil Plan-Apochromat objective. Image acquisition and processing were achieved using the Zeiss Efficient Navigation (ZEN) software. The images shown are the maximum-intensity projections of optical sections acquired at 0.5 µm.

Fixation and immuno-staining of larval brains was performed as previously described [S8]. Brain squashes were incubated overnight at 4°C with the following primary antibodies: rabbit anti DSpd-2 [S9], monoclonal anti-α-Tubulin (1:1,000; Sigma-Aldrich), rat anti-TCP-1α (Abcam) and rabbit anti-GFP (1:300; Torrey Pines Biolabs), which were detected by 1 hour incubation at room temperature with fluorescein isothiocyanate (FITC)-conjugated anti-mouse IgG+IgM (1:20; Jackson Laboratories), CY3-coniugated anti-rabbit IgG (1:300; Invitrogen), FITC-coniugated anti-rabbit IgG (1:50; Jackson Laboratories) and FITC-conjugated anti-rat IgG (1:20; Roche). Immunostained

preparations were mounted in Vectashield medium H-1200 (Vector Laboratories) containing the DNA dye DAPI, and examined with a Zeiss Axioplan fluorescence microscope equipped with a CCD camera (Photometrics CoolSnap HQ).

Microtubule regrowth assay

Larval brains were dissected in 0.7% NaCl and placed on ice for 50 min. After cold-induced depolymerisation, brains were either immediately fixed as previously described [S8], or placed at 22°C for 1, 3, 5 min and then fixed. All preparations were immunostained for α-Tubulin and Dspd-2 as described above.

Size exclusion chromatography

Analytical gel filtration chromatography was carried out using a Superose 6 10/300 GL column (24 ml bed volume; GE Healthcare) attached to an AKTA pure 25L system (Life Tech). Columns were run with C Buffer (without protease inhibitors) at 4°C at a flow of 0.5 mL/min. The following globular molecular weight standards were used to calibrate the column, at 10mg/ml: Dextran 2000 (2 MDa), thyroglobulin (669 kDa), apoferritin (440 kDa), alcohol dehydrogenase (DH) (150 kDa), albumin (67 kDa), carbonic anhydrase (29 kDa) (Sigma). The logarithm of the molecular weight (LogMW) of the standard proteins obtained from three independent runs was plotted against the elution volume (mL). 60 3rd instar larvae (Oregon-R, *mst* mutant and *Tcp1-α* RNAi) were flash frozen in N_2 (l) and stored at -80°C. Samples were ground using a pestle and mortar, suspended in 1 ml cold C buffer and spun at 65,000 rpm in

a TLA100 rotor in a Beckman Ultracentrifuge at 4°C for 30 min. Clarified supernatants were assessed for protein concentration using a nanodrop. Concentrations were: Oregon-R, 15.5 mg/ml; *mst*, 13.8 mg/ml; *Tcp-1*α RNAi, 13.6 mg/ml). 500 µl of clarified supernatant of each genotype was run sequentially through the column and 0.5 ml fractions collected. Aliquots were prepared for analysis by SDS-PAGE and western blotting by the addition of 6X protein sample buffer (PSB).

Larval MT sedimentation and tubulin stability assays

For the tubulin stability assay, batches of 30 control (Oregon-R) or *mst* 3rd instar larvae, flash frozen in N_2 (l) and stored at -80°C, were ground using a pestle and mortar, suspended in 500 µl of cold C buffer and spun at 65,000 rpm in a TLA100 rotor in a Beckman Ultracentrifuge at 4°C for 30 min. Immediately following centrifugation, 50 µl of extract was added to 10 µl of 6X PSB, while the remainder of the extract was placed in a water bath at 25°C. After 20 min, 40 min and 60 min, further 50 µl samples were added to 10 µl 6X PSB. Samples were run on standard SDS-PAGE gels and analysed by Western Blotting.

For MT sedimentation assays, clarified supernatants were generated from 30 frozen control or *mst* 3rd instar larvae as above. 150 µl of each clarified supernatant was incubated at 25°C for 20 min in the presence of 1 mM GTP, to stimulate MT polymerisation. Samples were carefully loaded over a two-volume cushion of C buffer + 40% glycerol and centrifuged at 65,000 rpm at 25°C for 12 min. 50 µl of supernatants were added to 10 µl of 6X PSB. The remaining supernatant was carefully removed, the interface between

supernatant and glycerol cushion washed with 100 µl of C Buffer, and all sample removed, being careful not to disturb any pellet. 50 µl of 1X PSB was added to the bottom of the tube, and pipetted to resuspend any pellet. This assay was undertaken on three separate occasions and produced qualitatively similar results in all cases, though the amount of Tubulin present in the *mst* mutant extracts, in relation to controls, varied, presumably due to variation in Tubulin degradation.

Homology search and MSA building

The homology search was performed on the Protein Data Bank database using HHsearch [S10], with HHblits [S11] MSA generation method (global alignment, secondary structure scoring, MSA1). Strong hits for models in the Tubulin and FtsZ families, derived from X-ray crystallography, with sequence coverage of over 60%, and resolution better than 3.2Å, were divided into individual domains (GTPase domain - MSA2 and activation domain - MSA3), and structurally realigned with POSA [S12] to account for flexibility of different conformational states. Using helix 7 as the overlapping region between the domains, the alignments (MSA2 and 3) were merged and manually refined (MSA4). Furthermore, we used HMMER (jackhammer) [S13] to find close homologues of *D. melanogaster* Mst in both invertebrates and vertebrates (NCBI nr database, MSA5). Hits covering more than 80% of the sequence were realigned using MAFFT [S14] (G-INS-i method, MSA6). In order to accurately relate Mst to tubulin/FtsZ, we merged the structural alignment of tubulin/FtsZ (MSA4) and the close homology alignment of Mst proteins (MSA6) using MAFFT [S15] (E-INS-i method, MSA7) while preserving both

alignments in their respective profiles.

Comparative modelling

Modelling of Mst was performed with Rosetta 3.5 [S16] comparative modelling protocol [S17]. The calculation was guided by the multiple sequence alignment (MSA7, Figure S4) to homologous proteins of known structure, minimising the conformational search space by providing a scaffold for protein backbone modelling. The structural templates used were: Tubulins: 4I4T A, 4I4T B, 3CB2 A; Bacterial tubulins (Btub): 2BTO A, 2BTQ B; CetZ: 3ZID A, 4B45 A, 4B46 A**;** FtsZ: 2R75 1, 2VXY A, 4M8I A, 1RQ2 A, 1OFU A, 2VAW A, 2VAP A, 1W5F A/B (GTPase domain from A, and activation domain from B); Bacterial TubZ: 3M89 A, 4EI7 A**;** Bacteriophage TubZ: 3V3T A, 3ZBQ A, 3R4V A. RosettaCM uses hybridisation protocol which allows for simultaneous sampling of multiple conformations featured in various protein templates. Regions considerably divergent from templates or those involving insertions or 'loops', were modeled *de novo* with Rosetta loop-building protocol. In order to exhaustively sample the local conformational space of the loops exhaustively, we calculated 2000 decoys: 400 decoys for each of the 5 best scoring templates (PDB ID: 2BTO_A, 2BTQ_B, 3CB2_A, 4I4T_A, 4I4T_B), which were further refined using Rosetta all-atom scoring function, energetically minimising the entire protein. The decoys were subsequently clustered with CALIBUR [S18] to identify structurally similar models.

Other bioinformatics and structural calculations

Coiled-coil predictions were carried out using COILS [S19] (windows: 14, 21

and 28; MTK matrix), Marcoil [S20] (MTK matrix) and Multicoil2 [S21] (Figure S5). Protein disorder was estimated using PrDOS [S22] and DISOPRED2 [S23], which was also used to predict propensity of disordered regions to bind globular proteins. Protein structure figures were prepared in Chimera [S24], which was also used to calculate superimpositions (MatchMaker tool).

SUPPLEMENTARY REFERENCES

S1. Miklos, G.L., Yamamoto, M., Burns, R.G., and Maleszka, R. (1997). An essential cell division gene of *Drosophila*, absent from *Saccharomyces*, encodes an unusual protein with tubulin-like and myosin-like peptide motifs. Proc. Natl. Acad. Sci. U S A. 94, 5189-94.

S2. Ripoll, P., Casal, J., and Gonzalez, C. (1987). Towards the genetic dissection of mitosis in *Drosophila*. BioEssays. 7: 204-210.

S3. Delgehyr, N., Wieland, U., Rangone, H., Pinson, X., Mao, G., Dzhindzhev, N.S., McLean, D., Riparbelli, M.G., Llamazares, S., Callaini, G., et al. (2012). *Drosophila* Mgr, a Prefoldin subunit cooperating with von Hippel Lindau to regulate tubulin stability. Proc. Natl. Acad. Sci. U S A. 109, 5729-34.

S4. Dietzl, G., Chen, D., Schnorrer, F., Su, K.C., Barinova, Y., Fellner, M., Gasser, B., Kinsey, K., Oppel, S., Scheiblauer, S., et al. (2007). A genome-wide transgenic RNAi library for conditional gene inactivation in *Drosophila*. Nature. 448, 151-6.

S5. Suster, M.L., Seugnet, L., Bate, M., and Sokolowski, M.B. (2004). Refining GAL4-driven transgene expression in *Drosophila* with a GAL80 enhancer-trap. Genesis. 39, 240-5.

S6. Rahmani, Z., Gagou, M.E., Lefebvre, C., Emre, D., and Karess, R.E. (2009). Separating the spindle, checkpoint, and timer functions of BubR1. J. Cell Biol. 187, 597-605.

S7. Giansanti, M.G., Bonaccorsi, S., Kurek, R., Farkas, R.M., Dimitri, P., Fuller, M.T., and Gatti, M. (2006). The class I PITP giotto is required for

Drosophila cytokinesis. Curr. Biol. 16, 195-201.

S8. Bonaccorsi, S., Giansanti, M.G., and Gatti, M. (2000). Spindle assembly in *Drosophila* neuroblasts and ganglion mother cells. Nat. Cell Biol. 2, 54-6.

S9. Giansanti, M.G., Bucciarelli, E., Bonaccorsi, S., and Gatti, M. (2008). *Drosophila* SPD-2 is an essential centriole component required for PCM recruitment and astral-microtubule nucleation. Curr. Biol. 18, 303-9.

S10. Söding, J. (2005). Protein homology detection by HMM-HMM comparison. Bioinformatics. 21, 951-60.

S11. Remmert, M., Biegert, A., Hauser, A., and Söding, J. (2011). HHblits: lightning-fast iterative protein sequence searching by HMM-HMM alignment. Nat. Methods. 9, 173-5.

S12. Li, Z., Natarajan, P., Ye, Y., Hrabe, T., and Godzik, A. (2014). POSA: a user-driven, interactive multiple protein structure alignment server. Nucleic Acids Res. 42, W240-5.

S13. Finn, R.D., Clements, J., and Eddy, S.R. (2011). HMMER web server: interactive sequence similarity searching. Nucleic Acids Res. 39, W29-37.

S14. Katoh, K., and Standley, D.M. (2013). MAFFT multiple sequence alignment software version 7: improvements in performance and usability. Mol Biol Evol. 30, 772-80.

S15. Katoh, K., and Frith, M.C. (2012). Adding unaligned sequences into an existing alignment using MAFFT and LAST. Bioinformatics. 28, 3144-6.

S16. Leaver-Fay, A., Tyka, M., Lewis, S.M., Lange, O.F., Thompson, J., Jacak, R., Kaufman, K., Renfrew, P.D., Smith, C.A., Sheffler, W., et al. (2011). ROSETTA3: an object-oriented software suite for the simulation and design of macromolecules. Methods Enzymol. 487, 545-74.

S17. Thompson, J., and Baker, D. (2011). Incorporation of evolutionary information into Rosetta comparative modeling. Proteins. 79, 2380-8.

S18. Li, S.C., and Ng, Y.K. (2010). Calibur: a tool for clustering large numbers of protein decoys. BMC Bioinformatics, 11:25.

S19. Lupas, A., Van Dyke, M., and Stock, J. (1991). Predicting coiled coils from protein sequences. Science. 252, 1162-4.

S20. Delorenzi, M., and Speed, T. (2002). An HMM model for coiled-coil domains and a comparison with PSSM-based predictions. Bioinformatics. 18, 617-25.

S21. Trigg, J., Gutwin, K., Keating, A.E., and Berger, B. (2011). Multicoil2: predicting coiled coils and their oligomerization states from sequence in the twilight zone. PLoS One. 6, e23519.

S22. Ishida, T., and Kinoshita, K. (2007). PrDOS: prediction of disordered protein regions from amino acid sequence. Nucleic Acids Res. 35, W460-4.

S23. Ward, J.J., McGuffin, L.J., Bryson, K., Buxton, B.F., and Jones, D.T. (2004). The DISOPRED server for the prediction of protein disorder. Bioinformatics. 20, 2138-9.

S24. Pettersen, E.F., Goddard, T.D., Huang, C.C., Couch, G.S., Greenblatt, D.M., Meng, E.C., and Ferrin, T.E. (2004). UCSF Chimera--a visualization system for exploratory research and analysis. J Comput Chem. 25, 1605-12.
